# Rapid Synthesis and Microenvironment Optimization of Hierarchical Porous Fe─N─C Catalysts for Enhanced ORR in Microbial Fuel Cells

**DOI:** 10.1002/advs.202402610

**Published:** 2024-06-17

**Authors:** Bolong Jiang, Nan Jiang, Yanyan Cui, Huan Wang, Geng Zhang, Jiayou Li, Yuhan Zhang

**Affiliations:** ^1^ Innovation Institute for Sustainable Maritime Architecture Research and Technology Qingdao University of Technology Qingdao Shandong 266000 China; ^2^ Institute of Environmental and Municipal Engineering Qingdao University of Technology Qingdao Shandong 266000 China; ^3^ College of Chemistry and Chemical Engineering Northeast Petroleum University Daqing Heilongjiang 163318 China

**Keywords:** Fe─N─C, hierarchical porous, microbial fuel cells, microenvironment, oxygen reduction reaction

## Abstract

Here, an approach to produce a hierarchical porous Fe‐N‐C@TABOH catalyst with densely accessible high intrinsic active FeN*_x_* sites is proposed. The method involves a single‐step pyrolysis of Zn/Fe‐zeolitic imidazolate framework (Zn/Fe‐ZIF‐H) with tetrabutylammonium hydroxide (TABOH) micelles, which is obtained by utilizing TABOH as a structural template and electronic mediator at room temperature for a brief duration of 16 min. Notably, the yield of Zn/Fe‐ZIF‐H is 3.5 times that of Zn/Fe‐ZIF‐N prepared by conventional method. Results indicate that in addition to expediting synthesis and increasing yield of the Zn/Fe‐ZIF‐H, the TABOH induces a hierarchical porous structure and fosters the formation of more and higher intrinsic active FeN*_x_* moieties in Fe*_x_*‐N‐C@TABOH, showing that TABOH is a multifunctional template. Crucially, the increased mesoporosity/external surface area and optimized microenvironment of Fe‐N‐C@TABOH significantly enhance ORR activity by facilitating the formation of high intrinsic active FeN*_x_* sites, increasing accessible FeN*_x_* sites, and reducing mass transfer resistance. Through structure tailoring and microenvironment optimization, the resulting Fe‐N‐C@TABOH exhibits superior ORR performance. DFT calculation further validates that the synergistic effect of these two factors leads to low ORR barrier and optimized ^*^OH adsorption energy. This study underscores the importance of structure and electronic engineering in the development of highly active ORR catalysts.

## Graphical abstract

A simple rapid strategy to fabricate a hierarchical porous Fe‐N‐C@TABOH catalyst with densely accessible high intrinsic active FeN_x_ sites is proposed. Benefit from structure tailoring and microenvironment optimization, the resulting Fe‐N‐C@TABOH showed superior ORR performance, offering an exceptionally high MFC power density of 830.1 ±5.2 mW·m^–2^, which is comparable to that of the Pt/C catalyst.

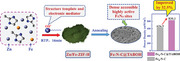

## 1 Introduction

Environmental pollution and energy shortages are two major problems facing humanity.^[^ ^1^ ^]^ Microbial fuel cells (MFCs) are novel environmentally friendly devices that use microorganisms as biocatalysts to convert soluble organic matter into electric energy.^[^ ^2^ ^]^ Nevertheless, the slow kinetics of the oxygen reduction reaction (ORR) on the cathode presents a significant challenge that hampers power generation in MFCs.^[^ ^3^ ^]^ Pt‐based catalysts generally are typically effective for ORR. However, their high cost and limited availability pose major barriers to the widespread adoption of MFCs in the energy sector.^[^ ^4^ ^]^ Consequently, there is a pressing need for the development of cost‐effective and highly efficient ORR electrocatalysts.^[^ ^5^ ^]^

Nitrogen‐doped carbon (NC) supported iron catalysts (Fe─N─C) have demonstrated significant promise as materials for ORR due to their cost‐effectiveness, eco‐friendliness, and favorable ORR performance.^[^ ^6^ ^]^ To date, numerous efforts have been devoted to synthesizing Fe─N─C catalysts.^[^ ^7^ ^]^ Among these approaches, the pyrolysis of Fe‐doped ZIF‐8 precursors is regarded as one of the most direct and efficient methods, attributed to the pre‐existing uniformly distributed metal‐nitrogen coordinated environment and the high surface area of ZIF‐8.^[^ ^8^ ^]^ Zn can isolate doped metal atoms, while the volatilization of Zn during pyrolysis can induce voids and form abundant defect sites.^[^ ^9^ ^]^ Therefore, ZIF‐8 is an ideal active site host for Fe as well as C and N precursors because of its high N content and low Zn center boiling point. Generally, ZIF‐8 can be prepared in organic solvents such as dimethylformamide (DMF) or methanol under thermal conditions. Furthermore, several groups have also reported methods for preparing ZIF‐8 in the water phase at moderate temperatures. However, the above methods are time‐consuming, and the yield of ZIF‐8 is low, resulting in a high preparation cost. Therefore, there is an urgent need to develop an alternative strategy for the efficient synthesis of ZIF‐8.

The FeN*_x_* sites within Fe─N─C catalysts are known as active sites for the ORR. The arrangement of FeN*_x_* significantly impacts the electronic state of the Fe center, thereby influencing its inherent ORR activity. Importantly, the method and conditions used for preparation are critical factors affecting the formation and utilization of active FeN*_x_* sites. The catalytic Fe–N*_x_* site is a molecular assembly that connects the two‐pore walls. During the high‐temperature treatment, the micropores with short wall length are conducive to bridge the two pore walls to form Fe–N*_x_* active sites.^[^ ^10^ ^]^ Therefore, Micropores, particularly well‐organized ones found in ZIF‐8, are vital for FeN*_x_* site formation. However, solely relying on microporous materials can lead to ORR limitations due to mass transfer issues.^[^ ^11^ ^]^ FeN*_x_* sites located near the external surface of micropores are easily accessible to reactants and contribute to ORR, whereas those buried deep within the dense carbon matrix of micropores make minimal contributions due to mass transfer resistance.^[^ ^12^ ^]^ Therefore, in addition to increasing the density of FeN*_x_* sites by creating micropores, building hierarchically porous structures by intruding mesopores/macropores is important for improving the utilization of FeN*_x_* sites.^[^ ^13^ ^]^ Mesopores (between 4 and 6 nm) can increase the accessible surface area with a lower transport resistance, while large mesopores (between 10 and 50 nm) and macropores (>50 nm) can act as reactant buffering reservoirs to shorten transport pathways and reduce electron transport resistance.^[^ ^14^ ^]^ Unfortunately, the catalysts obtained by direct pyrolysis of ZIF‐8 prepared by traditional methods are usually dominated by micropores.^[^ ^15^ ^]^ Shang et al.^[^ ^16^ ^]^ developed ZIF‐derived Co,N‐Co‐doped carbon nanoframes via mesoporous‐silica‐protected pyrolysis calcination, necessitating the use of highly corrosive and toxic hydrofluoric acid to eliminate the silica shell. Meanwhile, Wu et al.^[^ ^17^ ^]^ introduced a method utilizing the cationic surfactant cetyltrimethylammonium bromide (CTAB) and the amino acid L‐histidine (His) as co‐templates to fabricate ZIF‐8 with mesoporous diameters ranging from 4 to 20 nm for effective arsenate removal. However, this synthesis process is characterized by its time‐consuming nature, low yield, and high cost. Besides tailoring pore sizes, effectively modulating the microenvironment of central Fe is crucial for generating high intrinsic active FeN*_x_* sites. In conclusion, there is an urgent need for a straightforward and efficient methodology to prepare hierarchical porous ZIF‐8 while simultaneously engineering the electronic properties of central Fe, aiming to produce highly active Fe─N─C ORR catalysts.^[^ ^18^ ^]^

Here, we present a straightforward and effective method for the rapid fabrication of a hierarchical porous Fe‐N‐C@TABOH catalyst with densely accessible high intrinsic ORR active FeN*_x_* sites. The preparation entails the direct pyrolysis of Zn/Fe‐ZIF‐H with TABOH micelles, obtained by utilizing TABOH as a template and electronic mediator at room temperature within a remarkably short duration of 16 min, which greatly improved the preparation efficiency and increased the yield. The results revealed that during pyrolysis, in addition to generating micro, meso‐ and macropores by eliminating the micelles, nitrogen‐containing TABOH also acted as an electronic mediator to optimize the charge distribution of the Fe centers by providing additional nitrogen; thus, promoting the formation of dense highly intrinsically active FeN*_x_* moieties, as evidenced by X‐ray absorption near edge structure (XANES) and Mössbauer spectroscopy analysis. More importantly, the mesoporosity/external surface area and microenvironment optimization play a crucial role in boosting the ORR activity by promoting the formation of highly intrinsically active FeN*_x_* sites, increasing the number of accessible FeN*_x_* sites, and decreasing the mass transfer resistance. Benefiting from structure tailoring and microenvironment optimization, the fabricated Fe‐N‐C@TABOH catalyst demonstrated superior ORR performance. DFT calculations also confirmed that the synergistic effect of these two factors leads to a low ORR barrier and weak ^*^OH adsorption energy. This work provides an effective route for constructing highly active transition metal Fe‐based ORR catalysts.

## 2 Results and Discussion

### 2.1 Synthesis and Physicochemical Characterization of Fe*_x_*‐N‐C@TABOH

**Figure 1:**
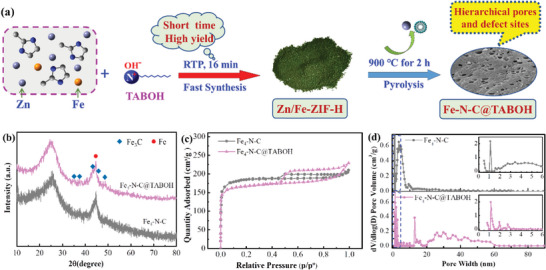
a) Synthesis, b) XRD patterns, c) N_2_ adsorption–desorption isotherms and d) pore size distributions of Fe*_x_*‐N‐C@TABOH and Fe_4_‐N‐C.

Briefly, 2‐methylimidazole (2‐MI) and zinc acetate (C_4_H_6_O_4_Zn) and FeSO_4_∙7H_2_O with varying molar contents of Fe are dissolved in CH_3_OH and thoroughly stirred at room temperature (**Figure** **1**a). TABOH is then added and stirred for 16 min. During this process, TABOH micelles are formed, and 2‐MI is rapidly deprotonated by OH^−^ on the surface of the micelles, leading to the formation of activated 2‐MI. The activated 2‐MI coordinates with Zn^2+^ and Fe^2+^ on the micelle surface, resulting in the rapid self‐assembly of Zn/Fe_4_‐ZIF‐H. Subsequently, the Zn/Fe_4_‐ZIF‐H precursors are annealed in an argon atmosphere to eliminate the TABOH micelles to obtain Fe_4_‐N‐C@TABOH with Fe content of 4.325 wt.%, thereby modifying the pore structure and producing a hierarchical porous material. For comparison, Fe_4_‐N‐C with Fe content of 5.92 wt.% was prepared through pyrolysis Zn/Fe_4_‐ZIF‐N obtained by conventional method. Notably, the yield of Zn/Fe‐ZIF‐H is 3.5 times that of Zn/Fe‐ZIF‐N, indicating that the sample yield improved after using TABOH.

The crystalline structure of the samples was evaluated by X‐ray diffraction (XRD) analysis (Figure 1b; Figure S2, Supporting Information). The strong broad peak ≈2*θ* =  25.3° corresponds to graphitic carbon (JCPDS NO. 13–0148). The graphitic carbon peak in Fe_4_‐N‐C@TABOH shifted to a small angle relative to Fe_1_‐N‐C@TABOH, indicating more nitrogen atoms are incorporated (Figure 1b). A typical strong peak of Fe^0^ (PDF#06‐0696) is observed at 2*θ* =  44.6°, indicating that Fe^0^ is the main phase of Fe*_x_*‐N‐C@TABOH and Fe_4_‐N‐C. In addition, with increasing Fe content *x*, the strength of the peak increases. The weak peaks at 2*θ* = 35.2°, 37.6°, 43.7°, 45.8° and 48.6° can be assigned to Fe_3_C (PDF#35‐0772),^[^ ^13^ ^]^ demonstrating that the content of Fe_3_C species is low.

The surface area and porosity of the samples are shown in Figure 1c,d, and Figure S3 and Table S1 (Supporting Information). Fe_4_‐N‐C is dominated by type I isotherms with a small hysteresis loop (Figure 1c), which is characteristic of microporous materials. All the isotherms of Fe*_x_*‐N‐C@TABOH are identified as combinations of type I and type IV with H4 hysteresis loops (Figure 1c; Figure S3a, Supporting Information),^[^ ^13^ ^]^ indicating the coexistence of micropores and mesopores in these samples.^[^ ^18^ ^]^ As depicted in Figure 1d and Figure S3b–d (Supporting Information), for *x  =  *1–4, the pores are concentrated at 13.1 nm and 21–63 nm, respectively. This confirms the hierarchically porous micropore/mesopore/macropore structure of Fe*_x_*‐N‐C@TABOH. It is well known that introducing mesopores/macropores to microporous materials is highly important for increasing the utilization of FeN*_x_* sites. The pore size distribution becomes wider with further increases in the Fe content (Figure S3d, Supporting Information), possibly because of partial channel blockage by excess iron. Micropores are essential for hosting more active sites, while mesopores can promote fast mass/charge transfer and facilitate the access of reactants to internal active sites, which can enhance ORR activity by increasing the accessibility of FeN*_x_* sites.^[^ ^19^ ^]^ Among the Fe*_x_*‐N‐C@TABOH catalysts, Fe_4_‐N‐C@TABOH had the highest *V*_meso_ (0.39 cm^3^·g^−1^) (Table S1, Supporting Information). The surface area of Fe_4_‐N‐C is 516 m^2^·g^−1^, which is similar to that obtained by Lai et al.^[^ ^20^ ^]^ Significantly, with increasing Fe content, *S*_external_ first increased and then decreased, reaching a maximum value at *x = *4% (248.0 m^2^·g^−1^) (Table S1, Supporting Information), which is in line with the trend of *V*_meso_. The FeN*_x_* sites located near the external surface are easily accessible to reactants. Therefore, a higher *S*_external_ facilitates the formation of active sites that can participate in the reaction. Moreover, Fe_4_‐N‐C@TABOH exhibited a much greater *S*_external_ and *V*_meso_ than did Fe_4_‐N‐C. The above results reveal that the high *S*_external_ and abundant mesopores of Fe_4_‐N‐C@TABOH originate from the use of the TABOH template and the introduction of an appropriate amount of Fe.

**Figure 2:**
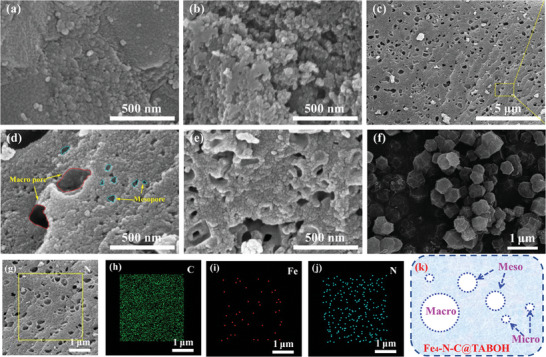
SEM images of a) Fe_1_‐N‐C@TABOH, b) Fe_2_‐N‐C@TABOH, c,d,k) Fe_4_‐N‐C@TABOH, e) Fe_8_‐N‐C@TABOH, f) Fe_4_‐N‐C; g–j) Mapping image of Fe_4_‐N‐C@TABOH.

SEM images of Fe*_x_*‐N‐C@TABOH and Fe_4_‐N‐C are shown in **Figure** **2**. Fe_4_‐N‐C displays a uniform polyhedron structure (Figure 2f), which is in line with the morphology of ZIF‐8. Fe_1_‐N‐C@TABOH presents a random small spherical structure that aggregates together, while for Fe_2_‐N‐C@TABOH, some small holes can be observed. With a further increase in the Fe content, more holes appeared, and the surface became looser. Fe_4_‐N‐C@TABOH exhibited hierarchically structured mesopores with several large holes, which is consistent with the BET analysis. The mapping images of Fe_4_‐N‐C@TABOH suggest that Fe, N, and C are well scattered on the surface (Figure 2g–j).

**Figure 3:**
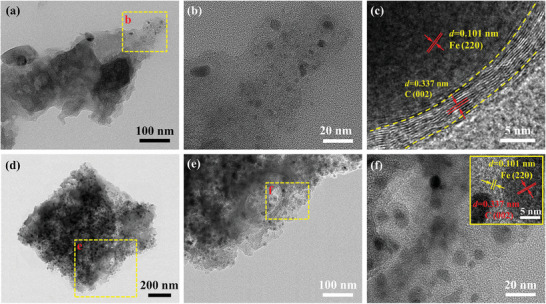
TEM analysis of a–c) Fe_4_‐N‐C@TABOH and d–f) Fe_4_‐N‐C.

The TEM images of Fe_4_‐N‐C@TABOH in **Figure** **3**a–c show that iron nanoparticles with an average size of 3.7 nm are uniformly dispersed on the N‐doped carbon matrix. Moreover, no obvious aggregation is observed. The high‐resolution TEM images confirmed that the Fe nanoparticles were successfully wrapped in the N‐doped carbon matrix (Figure 3c,f). The lattice spacing of 0.337 nm corresponds to the (002) crystal plane of carbon.^[^ ^21^ ^]^ The lattice spacing of 0.101 nm matches the (220) planes of Fe. The average iron nanoparticle size of Fe_4_‐N‐C (7.4 nm) is larger than that of Fe_4_‐N‐C@TABOH, showing that the use of TABOH leads to a smaller Fe particle size.

**Figure 4:**
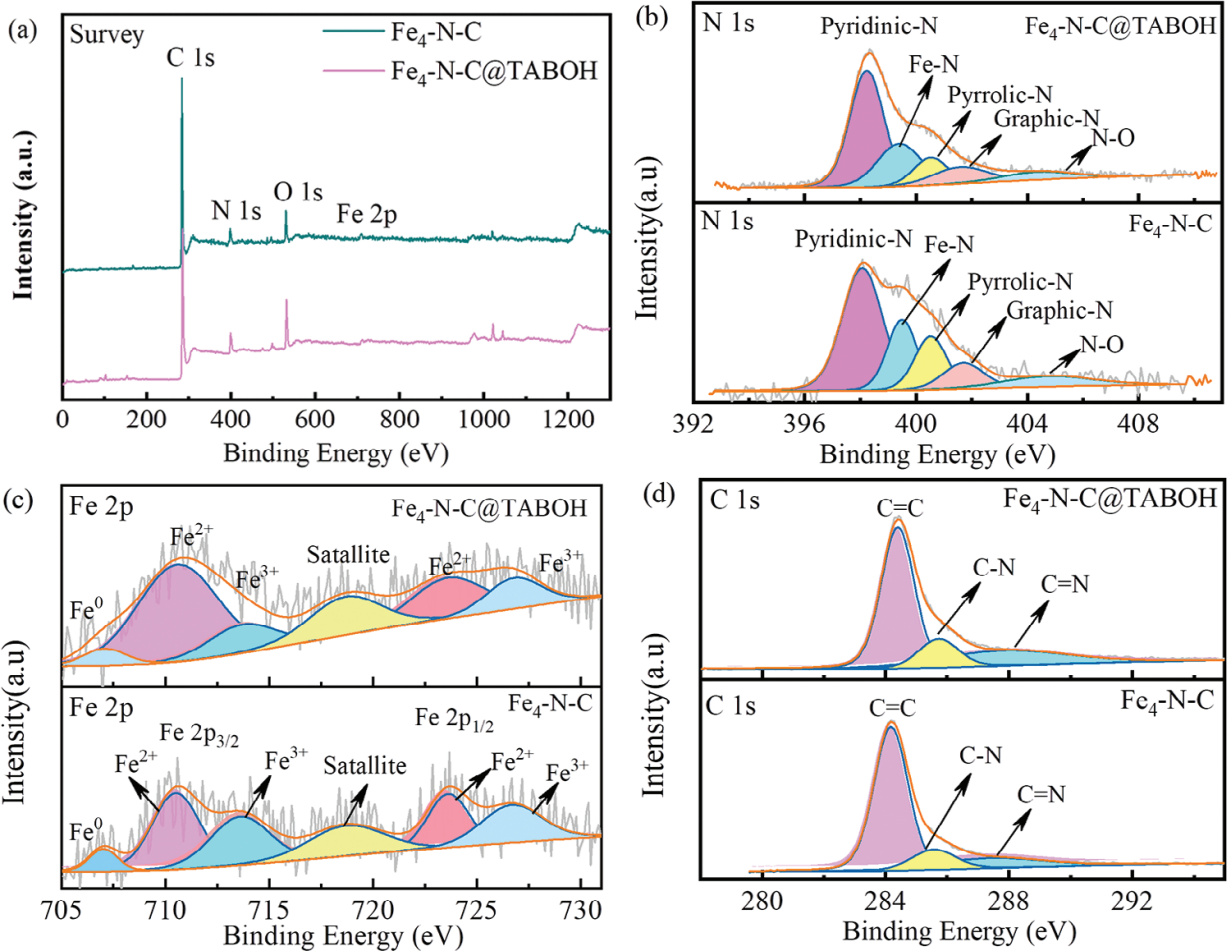
XPS spectra of a) Fe_4_‐N‐C@TABOH and Fe_4_‐N‐C of survey, b) N 1s, c) Fe 2p, and d) C 1s.

The chemical composition and electronic structure were studied by X‐ray photoelectron spectroscopy (XPS). The results in **Figures** **4**a and S4 (Supporting Information) show the presence of Fe, C, and N in all the catalysts. The N 1s signals (Figure 4b; Figure S5, Supporting Information) of the samples present mainly pyridinic N (398.2–398.4 eV), Fe‐N*_x_* (399.4–399.7 eV), pyrrolic N (400.3–400.6 eV), graphitic N (401.4–401.8 eV) and oxidic N (404.0–404.4 eV).^[^ ^22^ ^]^ Heteroatom N doping in a C matrix can alter the charge or spin distribution, which facilitates oxygen adsorption. Furthermore, the FeN*_x_* sites were proven to be ORR active sites. The total N content in all the Fe*_x_*‐N‐C@TABOH samples was greater than that in the Fe_4_‐N‐C sample, which is beneficial for coordinating with the metal sites, as directly evidenced by the relatively high FeN*_x_* content (Table S2, Supporting Information). Moreover, the N sites in the carbon matrix are beneficial for improving the adsorption of oxygen and promoting ion diffusion. Among all the samples, Fe_4_‐N‐C@TABOH (22.64%) has the highest Fe‐N*_x_* content, which is significantly greater than that of Fe_4_‐N‐C (19.45%). This further validates that the additional mesoporosity/external surface area facilitated by TABOH promotes the exposure of densely active Fe‐N*_x_* sites, consequently enhancing ORR performance. Significantly, Fe_4_‐N‐C@TABOH exhibits the highest graphitic N content of 11.13%, which facilitates electron transfer. The Fe 2p signals (Figure 4c) can be categorized into a peak of Fe^0^ (706.9 eV) and doublet peaks of Fe_2_^+^ (710.6 and 723.6 eV) and Fe^3+^ (713.7 and 726.8 eV).^[^ ^23^ ^]^ In the C 1s region (Figure 4d), the presence of C═C (284.4 eV), C═N (285.7 eV), and C═N (287.8 eV) bonds confirms the successful nitrogen doping into the carbon matrix.^[^ ^24^ ^]^ The O 1s signals (Figure S8, Supporting Information) reveals the presence of C─O bond (532.1 eV) and COOH group (531.4 eV) on the surface of the carbon matrix. The surface element content listed in Table S2 (Supporting Information) reveals that the Fe content in Fe*_x_*‐N‐C@TABOH ranges from 1.63–4.87 wt.%, which increases with increasing initial Fe content. Fe_4_‐N‐C has a greater surface Fe content (4.34 wt.%) than does Fe_4_‐N‐C@TABOH (3.33 wt.%), possibly because Fe_4_‐N‐C has a larger specific surface area than does Fe_4_‐N‐C@TABOH, and it is dominated by micropores since micropores are essential for the formation of iron sites. Although Fe_4_‐N‐C has a relatively high Fe content, its number of effective active sites can be affected by many factors. This will be discussed further in Section 3.5, which will be combined with the characterization technique. The Raman spectra in Figure S9 (Supporting Information) exhibit a D band (1355 cm^−1^) and a G band (1582 cm^−1^).^[^ ^25^ ^]^ Notably, compared with Fe_4_‐N‐C (3.27), Fe_4_‐N‐C@TABOH exhibits more structural defects and more abundant vacancies, as revealed by its greater *I*_D_/*I*_G_ value (33.4).^[^ ^26^ ^]^

### 2.2 Electrochemical Test

**Figure 5:**
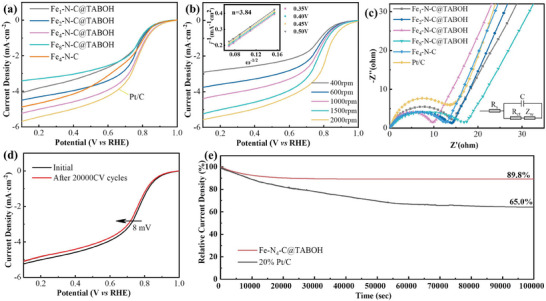
a) LSV plots for Fe*_x_*‐N‐C@TABOH and Pt/C, b) LSV and Koutecky–Levich plots of Fe_4_‐N‐C@TABOH, c) Nyquist plots of Fe*_x_*‐N‐C@TABOH, d) LSV plots of Fe_4_‐N‐C@TABOH before and after 20 000 potential cycles, e) chronoamperometric *i–t* plots of Fe_4_‐N‐C@TABOH and 20% Pt/C.

The electrochemical performance of Fe*_x_*‐N‐C@TABOH was evaluated using cyclic voltammetry (CV) in a 0.1 m PBS electrolyte (Figure S10, Supporting Information). All catalysts exhibit distinct reduction response peaks corresponding to the ORR. As anticipated, the Fe_4_‐N‐C@TABOH demonstrates a more positive reduction response peak at 0.57 V, indicating excellent ORR catalytic performance. Linear sweep voltammetry (LSV) curves of Fe*_x_*‐N‐C@TABOH catalysts (**Figure** **5**a; Table S3, Supporting Information) reveal that among Fe*_x_*‐N‐C@TABOH (0.82–0.86 V and 0.66–0.71 V vs RHE), Fe_4_‐N‐C@TABOH (0.86 and 0.71 V vs RHE) displays a more positive onset potential and higher half‐wave potential, comparable to that of commercial Pt/C (0.87 and 0.71 V vs RHE), as summarized in Table S3 (Supporting Information), and superior to other catalysts reported in literatures (Table S7, Supporting Information). Furthermore, the limiting current density of Fe_4_‐N‐C@TABOH (−5.23 mA∙cm^−2^) notably exceeds those of other samples (−3.40 to −4.50 mA∙cm^−2^), including Fe_4_‐N‐C (−4.85 mA∙cm^−2^). This may be because Fe_4_‐N‐C@TABOH possesses the highest *S*_external_, *V*_meso_, and evenly distributed FeN*_x_* active sites. A high *S*_external_ is beneficial for increasing the number of accessible active FeN*_x_* sites and improving their utilization, while a large *V*_meso_ and hierarchically porous structure promotes the mass/charge transfer process. The superior ORR performance of Fe_4_‐N‐C@TABOH over Fe_4_‐N‐C, further demonstrated that the *S*_external_, *V*_meso_, and hierarchically porous structures originating from the use of the TABOH template and the introduction of an appropriate amount of Fe to form FeN*_x_* active sites with an optimized microenvironment are very important for achieving excellent ORR catalytic performance. The double layer capacitances (*C*_dl_), which were proportional to the ECSA, were calculated using CV method (See FigureS11 and Table S4, Supporting Information). The calculated *C*_dl_ and ECSA of Fe_4_‐N‐C@TABOH are 6.62 mF cm^−2^ and 34.15 cm^2^, respectively, which are larger than those other references, indicating the fast kinetics and high utilization of the active sites. More importantly, the high intrinsic activity of Fe_4_‐N‐C@TABOH is also supported by LSV curves after ECSA normalization (Figure S12, Supporting Information). The ORR pathways were determined by K–L plots obtained from the LSV curves (rotation rate 400–2000 rpm) (Figure 5b). The average electron transfer number (*n*) of Fe_4_‐N‐C@TABOH was calculated to be 3.84, confirming that the ORR process over Fe_4_‐N‐C@TABOH approximately follows a four‐electron transfer route. This fact is further proved by the low H_2_O_2_ yield (2.27–3.76%) over the tested potential range (Figure S13a, Supporting Information). Compared to Fe_4_‐N‐C (0.13 e s^−1^), turnover frequency (TOF) value at 0.6 V of Fe_4_‐N‐C@TABOH (0.27 e s^−1^) is doubled, confirming an improved intrinsic activity because of using TABOH (Figure S13b, Supporting Information). As depicted in Figure 5d and Figure S14 (Supporting Information), Fe_4_‐N‐C@TABOH delivers a remarkable durability with slight decay in activity (≈8 mV) after 20 000 continuous potential cycles, which is superior to that of 20%Pt/C. The CA test presents a retention rate of 89.8% after continuous operation for 100 000 s (Figure 5e), surpassing the 20% Pt/C (65.0%). XRD and SEM analysis of spent Fe_4_‐N‐C@TABOH reveal that the crystal structure and morphology have little changed compared with the corresponding fresh one, which indicates that the catalyst has good durability (Figure S15, Supporting Information).

The results of EIS (Figure 5c) and the data obtained from fitting an equivalent circuit model are presented in Table S3 (Supporting Information). The electrochemical reaction rate is inversely correlated with Rct, indicating that lower Rct values correspond to faster reaction rates. The charge transfer resistance (*R*_ct_) of Fe*_x_*‐N‐C@TABOH ranges from 9.7 to 16.8 Ω, with Fe_4_‐N‐C@TABOH exhibiting the lowest electrical resistance. Notably, the electrical resistance of Fe_4_‐N‐C@TABOH surpasses that of Fe_4_‐N‐C (12.6 Ω) and even outperforms that of commercial Pt/C (20 wt.% Pt, 13.5 Ω). This can be attributed to the high *V*_meso_ and hierarchically porous structure of Fe_4_‐N‐C@TABOH, which ensures an optimal mass/electron transfer process by reducing the distance to the active site. Moreover, the abundant graphitic N content (Table S2, Supporting Information) facilitates electron transfer. Essentially, the surplus electrons of nitrogen in the delocalized π‐system contribute to low charge transfer resistance.

### 2.3 MFC Output Voltage Test

**Figure 6:**
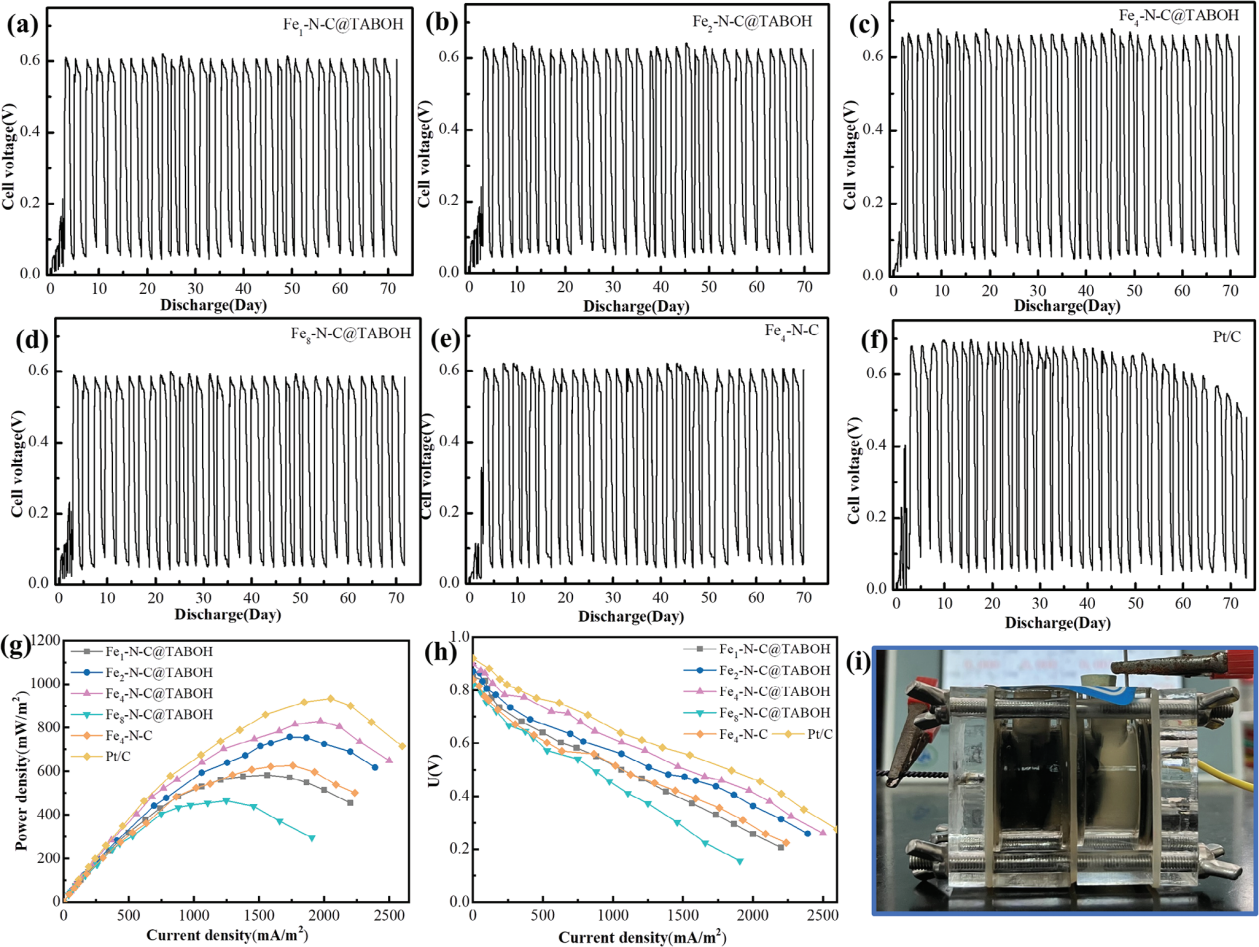
a–f) Output voltage curves of MFC, g) power density, h) cathode polarization curves of Fe*_x_*‐N‐C@TABOH, Fe_4_‐N‐C, and Pt/C.

**Figure 7:**
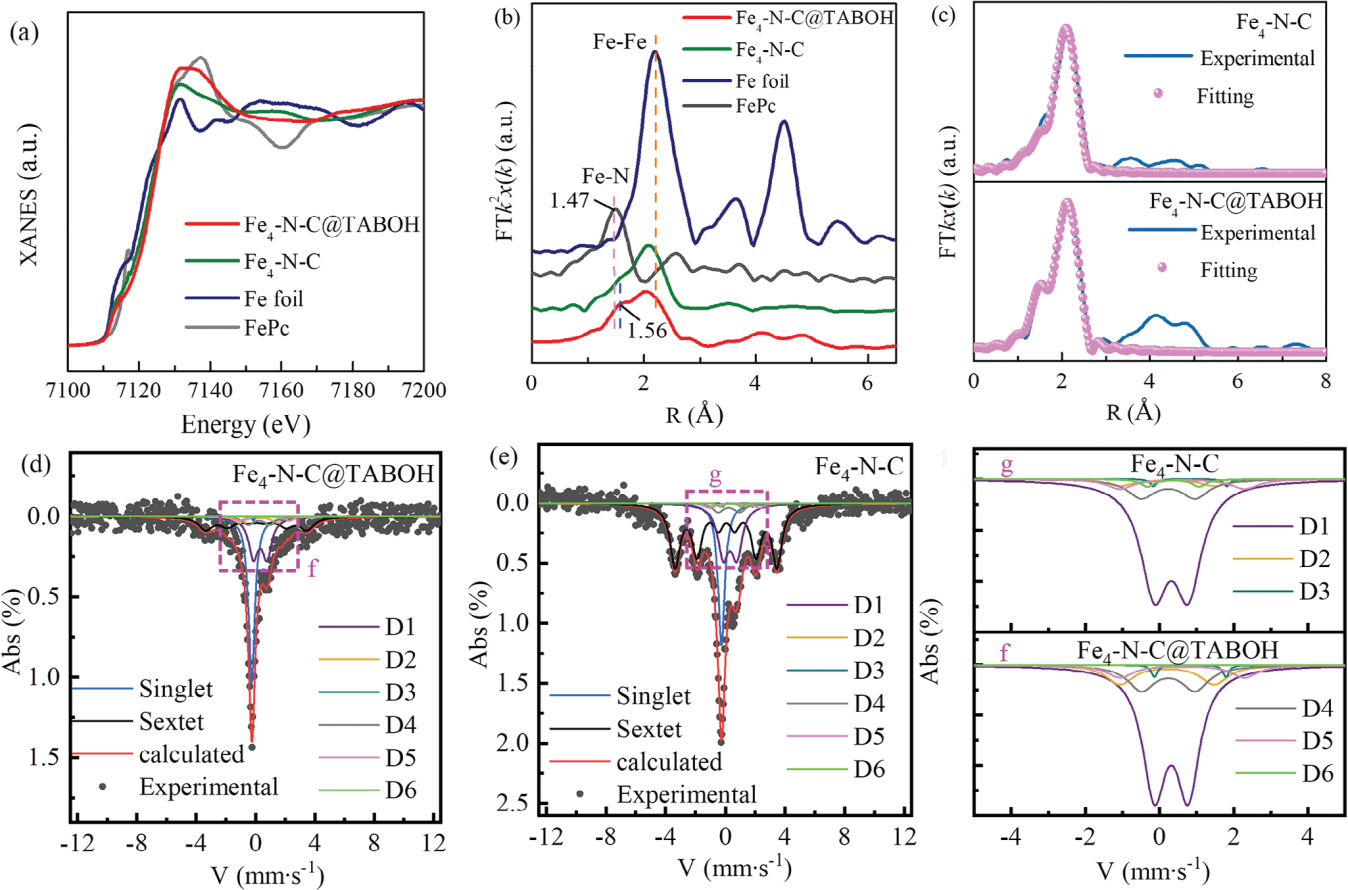
a) Fe K‐edge XANES spectra, b) k^2^‐weighted Fourier transform extended X‐ray absorption fine structure (EXAFS) spectra, c) EXAFS fitting curves in the R‐space of Fe_4_‐N‐C@TABOH and Fe_4_‐N‐C; d–g) ^57^Fe Mössbauer spectra of Fe_4_‐N‐C@TABOH and Fe_4_‐N‐C.

The continuous monitoring of the MFC output voltage over 75 days with a fixed 1000 Ω resistance revealed that Fe_4_‐N‐C@TABOH achieved the highest maximum output voltage of 0.67 V ±0.01 V, surpassing other Fe*_x_*‐N‐C@TABOH catalysts (ranging from 0.60 to 0.64 V) and comparable to commercial Pt/C (0.68 V ±0.03 V) (**Figure** **6**a–f). Throughout the 75‐day operation, MFCs equipped with Fe*_x_*‐N‐C@TABOH demonstrated notable stability with no significant decline in output voltage. However, MFCs employing commercial 20% Pt/C experienced a considerable decrease in output voltage to 0.47 V ±0.01 V after 75 days (**Figure** **7**f). Notably, the MFC output voltage of Fe_4_‐N‐C@TABOH exceeded that of Fe_4_‐N‐C by 11.5% (0.61 V ±0.02 V), underscoring the critical role of structure tailoring and microenvironment optimization using TABOH in enhancing ORR performance.

The power density presented in Figure 6g and Table S3 (Supporting Information) showed that among the Fe*_x_*‐N‐C@TABOH composites, Fe_4_‐N‐C@TABOH achieves the highest maximum power density of 830.1 ±5.2 mW∙m^−2^, significantly surpassing that of Fe_4_‐N‐C prepared without TABOH (626.5 ±4.0 mW∙m^−2^), albeit slightly lower than Pt/C (933.8 ±6.2 mW∙m^−2^). Importantly, the MFC power density of Fe_4_‐N‐C@TABOH is improved by 32.5% compare to that of Fe_4_‐N‐C (626.5 ±4.0 mW∙m^−2^) and superior to other catalysts reported in literatures (Table S8, Supporting Information). This enhancement stems from the reduced charge transfer resistance and superior ORR activity of Fe_4_‐N‐C@TABOH, attributed to the structure tailoring and microenvironment optimization enabled by TABOH. As depicted in Figure 6h, the open circuit voltage (OCV) of the MFC equipped with Fe_4_‐N‐C@TABOH reaches 0.90 V, the highest among those with Fe*_x_*‐N‐C@TABOH (0.85 to 0.90 V), and comparable to Pt/C (0.92 V). Moreover, the polarization curve of all electrodes reveals significant changes in cathode potential with increasing current density, indicating that differences in MFC power generation primarily arise from variations in cathode performance (Figure S16, Supporting Information).

### 2.4 Identification of the Electronic Properties of Central Fe and Theoretical Analysis

#### 2.4.1 Analysis of a Possible Synthetic Mechanism of Fe‐N‐C@TABOH

**Scheme 1:**
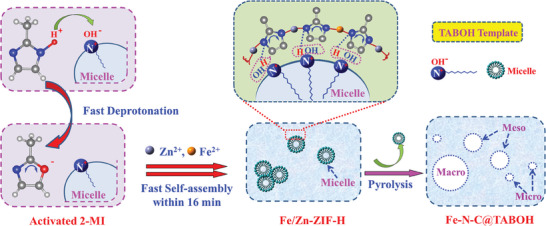
Possible mechanism of pore structure tailoring with TABOH.

To gain deeper insights into the structural tailoring effect of TABOH during the rapid synthesis of Fe/Zn‐ZIF‐H, the breaking energy (BE) of the H─N bond of 2‐methylimidazole (2‐MI) in the presence of TABOH template was computed. The BEs of the H─N bond in 2‐MI with and without TABOH are −26.2 and 1.9 kJ·mol^−1^, respectively (Figure S17, Supporting Information). This indicates that the deprotonation of 2‐MI can be significantly accelerated with the addition of the TABOH template. Furthermore, the order parameter *P* and the mesophase morphology during the synthesis system (where the blue M beads represent the methanol, the purple O beads represent the NOH segments, the green C beads represent the CH_4_ segments and the dark Z beads represent the ZIF precursors) were calculated using the MesoDyn module in Materials Studio software to explore the self‐assembly process. As depicted in Figure S18b_0_ (Supporting Information), *P* increases rapidly within 0.2 min, indicating the self‐assembly of TABOH to form micelles within a short time (Figure S18b_1_, Supporting Information). After 0.25 min, *P* drops sharply and then rises, suggesting that the system transitions to another ordered state, where chaotic micelles are gradually transformed into ordered micelles (Figure S18b_2_, Supporting Information). Subsequently, *P* stabilizes, indicating the formation of thermodynamically stable micelles (Figure S18b_3_, Supporting Information). Based on the above calculations, a possible mechanism is proposed (**Scheme** **1**): i) TABOH template self‐assembles to form micelles rapidly; ii) the H on the N─H of 2‐MI ring is attracted by OH^−^ on the outer surface of the micelles and quickly deprotonated to form activated 2‐MI; iii) the activated 2‐MI on the surface of micelles rapidly coordinates with Fe_2_^+^/Zn^2+^ to form the Fe/Zn‐ZIF‐H crystal skeleton; iv) the micelles are then removed by pyrolysis to generate Fe‐N‐C@TABOH with micro‐, meso‐, and macropores and abundant defect sites. In brief, TABOH promotes rapid deprotonation of 2‐MI and modulates the pore structure of samples.

To confirm the crystal phase structure of Zn/Fe_4_‐ZIF‐TABOH synthesized using the facile and rapid template strategy within a very short time, the XRD patterns of Zn/Fe_4_‐ZIF‐H and Zn/Fe_4_‐ZIF‐N are compared (Figure S19, Supporting Information). The peaks of Zn/Fe_4_‐ZIF‐N closely match those of a SOD type of ZIF‐8 structure. Moreover, the similarity of the XRD patterns and FTIR curve (Figure S20, Supporting Information) of Zn/Fe_4_‐ZIF‐H to those of Zn/Fe_4_‐ZIF‐N, Zn‐ZIF‐H, and Zn‐ZIF‐N confirms that the crystal structure and functional groups of Zn/Fe_4_‐ZIF‐H are not changed after using TABOH. The pore size distribution of Zn/Fe_4_‐ZIF‐N is primarily dominated by micropores, whereas Zn/Fe_4_‐ZIF‐H exhibits a hierarchical porous structure with micro‐, meso‐, and macropores (Figure S21, Supporting Information). The meso‐ and macropores are concentrated at 4.1, 33, and 57 nm, respectively. The SEM images reveal that Zn/Fe_4_‐ZIF‐N exhibits a uniform polyhedron structure (Figure S22, Supporting Information). However, the morphology of Zn/Fe_4_‐ZIF‐TABOH displays highly dispersed nanoparticles with very small particle sizes, which can be attributed to rapid nucleation within a short time in the presence of the TABOH template.

#### 2.4.2 Analysis of the Microenvironment of the Fe Center and the Formation Mechanism of FeNx Sites

To investigate the impact of TABOH on the local electronic properties of Fe atoms in the prepared catalyst, the X‐ray absorption near edge structure (XANES) of Fe_4_‐N‐C@TABOH was compared with that of Fe_4_‐N‐C. As illustrated in Figure 7a, the Fe‐edge adsorption energy of both Fe_4_‐N‐C@TABOH and Fe_4_‐N‐C catalysts is higher than that of Fe foil, indicating positively charged Fe atoms in both catalysts.^[^ ^27^ ^]^ FePc exhibits a peak at 7118.0 eV, corresponding to the typical characteristics of square‐planar D_4h_ local symmetry of Fe ion surrounded by four‐coordinated nitrogen ligands (FeN_4_).^[^ ^28^ ^]^ Similarly, both samples show a peak at 7118.0 eV. However, unlike Fe_4_‐N‐C, Fe_4_‐N‐C@TABOH displays an additional weak peak at 7114.6 eV, attributed to the square‐planar to five‐coordination (FeN_5_) system resulting from the additional binding of axial ligands.^[^ ^29^ ^]^

Additional structural insights can be derived from the Fourier transform of Fe K‐edge extended X‐ray absorption fine structure (FT‐EXAFS) results (Figure 7b). Both Fe_4_‐N‐C and Fe_4_‐N‐C@TABOH exhibit a peak of Fe‐N scattering path of FePc at 1.47 and 1.56 Å, respectively. Additionally, the peak observed at 2.08 Å (corresponding to the Fe–Fe ligands) can be attributed to the Fe atom. Furthermore, the WT contour plots (Figure S23a–d, Supporting Information) of Fe_4_‐N‐C@TABOH and Fe_4_‐N‐C samples also reveal an intensity maximum of the Fe‐N path similar to that of FePc.^[^ ^30^ ^]^ FT‐EXAFS fitting results (Figure 7c; Table S5, Supporting Information) indicate that Fe is coordinated with 4.8 nitrogen atoms with an average Fe─N bond‐length of 1.95 Å for Fe_4_‐N‐C@TABOH, while Fe_4_‐N‐C exhibits a coordination number of 4.0 with an average Fe─N bond‐length of 1.89 Å. The increase in coordination number of Fe with N suggests that nitrogen‐containing TABOH promotes the formation of some high intrinsic FeN_5_ active moiety structure in Fe_4_‐N‐C@TABOH, potentially enhancing the ORR activity. Notably, Fe_4_‐N‐C@TABOH presents a longer Fe─N bond length than Fe_4_‐N‐C (Table S5, Supporting Information), resulting in increased charge density of Fe due to electronic modulation from TABOH. This optimization benefits the adsorption/desorption of the ORR intermediates. These findings confirm a change in the microenvironment of the Fe center due to the use of TABOH electronic mediator, which will be further discussed later in this section.

**Figure 8:**
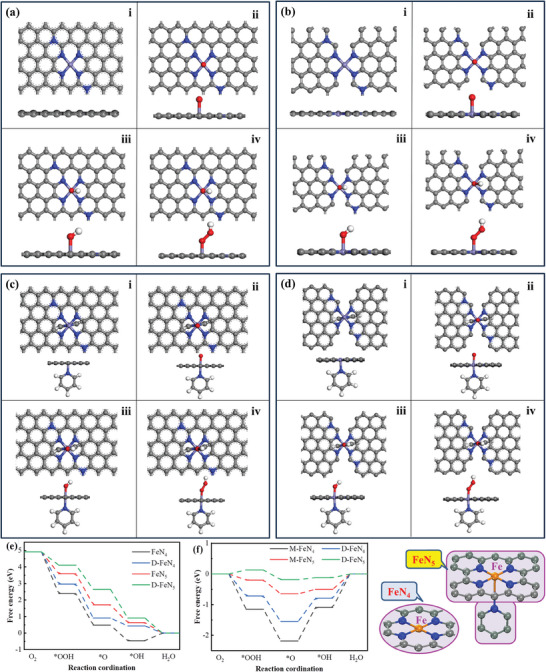
Optimized original structure (i) and ^*^O (ii), ^*^OH (iii), and ^*^OOH (iv) adsorbed on a) M‐FeN_4_, b) D‐FeN_4_, c) M‐FeN_5_ and d) D‐FeN_5_; Free‐energy diagrams of the reduction of O_2_ to H_2_O at e) 1.23 V and f) 0 V.

Mössbauer spectroscopy is employed to further elucidate the electronic state of active FeN*_x_* sites in Fe_4_‐N‐C@TABOH and Fe_4_‐N‐C. As depicted in the profiles in **Figure** **8**d–g, the spectra are deconvoluted into a sextet, singlet, and up to six doublets (D1–D6), with corresponding isomer shifts (IS) and quadrupole splitting (QS) values listed in Table S6 (Supporting Information). The sextets and singlet can be attributed to metallic iron or iron carbide. D1 (Fe^II^N_4_/C, low spin), D2 (Fe^II^N_4_/C, intermediate spin), and D3 (N‐Fe^II^N_2+2_/C, high spin) are associated with the FeN*_x_* centers exhibiting different electronic states.^[^ ^31^ ^]^ Additionally, D4, D5, and D6 can be assigned to Fe*_x_* N with *x* ≤ 2.1. Notably, the ORR activity primarily stems from D1 and D3 structures.^[^ ^32^ ^]^ This is because in the D1 and D3 structures, the 3d_z_^2^ orbitals of Fe^+2^ ions are not fully occupied, whereas D2 is fully occupied, thereby hindering the end‐on adsorption of molecular oxygen on these sites. D3, characterized by the highest activity site for the ORR, exhibits a turnover frequency two orders of magnitude higher than that of D1.^[^ ^30^ ^]^ This high intrinsic activity is attributed to i) the coordination of N with the Fe center of FeN_4_, which optimizes the electronic structure of Fe, and ii) the ability of the pyridinic N adjacent to the D3 site to quickly supply the required protons for the ORR.^[^ ^33^ ^]^ Table S6 (Supporting Information) shows that D1 and D3 in Fe_4_‐N‐C@TABOH accounted for 25.2% and 0.6%, respectively, which was higher than those in Fe_4_‐N‐C (21.5% and 0.3%). Crucially, the FeN_5_ sites (D3) in Fe_4_‐N‐C@TABOH is twice as abundant as that in Fe_4_‐N‐C, underscoring how the incorporation of TABOH can augment the presence of high intrinsic active FeN*_x_* moiety structures (D1 and D3). More importantly, coupled with the XANES findings, the distinct and indispensable role played by TABOH in electronically modulating the Fe center and fostering the formation of active FeN*_x_* sites is evident. During the high‐temperature heating process, the decomposition of TABOH micelles within Zn/Fe‐ZIF‐H yields meso‐ and macropores alongside defect sites. Concurrently, nitrogen atoms from TABOH micelles become embedded within the carbon matrix by providing additional nitrogen, with some forming FeN_4_ or FeN_5_ sites by coordinating with adjacent Fe atoms, thereby optimizing the microenvironment of the Fe center. This assertion finds support in the observed increase in total nitrogen content, as well as FeN_4_ and FeN_5_ site content following the use of TABOH (confirmed via XANES, Mössbauer spectroscopy, and XPS analysis), affirming the role of TABOH in electronic modulation. While FeN*_x_* sites are typically located in carbon micropores, those situated at the edge of such micropores exhibit higher activity compared to bulk‐hosted sites. Manipulating the pore structure to generate macropores and defect sites increases the proportion of edge carbon, thereby facilitating the formation of highly active sites. Thus, while micropores are pivotal for FeN*_x_* site formation, mesopores play a crucial role in facilitating the creation of highly active and accessible FeN*_x_* sites.

#### 2.4.3 Contribution of Different Active Sites

To evaluate the contributions of different active sites, Fe_0.5_‐N‐C@TABOH, Fe/Fe_3_C/C, and NC control catalysts are characterized and tested by electrochemical methods. As shown in Figure S24 (Supporting Information), XRD patterns of Fe_0.5_‐N‐C@TABOH and NC show two peaks at 25.3° and 43.2°, both of which can be assigned to graphitic carbon. There are no other detectable peaks of Fe or Fe_3_C in the XRD pattern of Fe_0.5_‐N‐C@TABOH, indicating that there is no or low content of Fe or Fe_3_C. For Fe/Fe_3_C/C, the typical strong peak of Fe^0^ (PDF#06‐0696) is observed at 2*θ* = 44.6° and 65.0°, demonstrating that Fe^0^ is the main phase. While the weak peaks at 2*θ* = 35.2°, 37.6 °, 43.6°, 44.9 °, 45.8 °, 48.6°, 56.0°, and 61.2°, can be assigned to Fe_3_C (PDF#35‐0772).^[^ ^13^ ^]^ The above results indicate the coexistence of Fe^0^ and Fe_3_C in Fe/Fe_3_C/C. In addition, SEM images of Fe/Fe_3_C/C (Figure S25a, Supporting Information), Fe_0.5_‐N‐C@TABOH (Figure S25b, Supporting Information), and NC (Figure S25c,d, Supporting Information) exhibited random spherical nanostructures. The TEM images of Fe/Fe_3_C/C in Figure S26a,b, (Supporting Information) further displays that the Fe nanoparticles are wrapped in N‐doped carbon matrix. In the high‐resolution TEM image (Figure S26c, Supporting Information), the lattice spacing of 0.337 nm corresponds to the (002) crystal plane of carbon, and the lattice spacing of 0.204 nm is related to the (220) plane of Fe_3_C or (110) plane of cubic Fe, respectively.^[^ ^34^ ^]^ Therefore, combined with the XRD results, the nanoparticles in Fe/Fe_3_C/C should be Fe/Fe_3_C nanocrystals. It is worth noting that no clear nanoparticles are seen in TEM images of Fe_0.5_‐N‐C@TABOH (Figure S26d–f, Supporting Information), which is possibly due to most of the Fe nanoparticles reacts with N to form uniformly dispersed FeN*_x_* sites at low Fe content, which is in line with XRD analysis. Similar result was also obtained by Jiang et al.^[^ ^35^ ^]^

The electrochemical performance of Fe_0.5_‐N‐C@TABOH, Fe/Fe_3_C/C, and NC was evaluated using linear sweep voltammetry (LSV) in a 0.1 m PBS electrolyte. LSV curve in Figure S27a (Supporting Information) reveals that the metal‐free NC (0.41 V vs RHE and −2.76 mA∙cm^−2^) presents a poor half‐wave potential and limiting current density. The ORR pathways were determined by K–L plots derived from the LSV curves (rotation rate 400–2000 rpm) (Figure S27, Supporting Information (inset)). The average electron transfer number (n) of NC is 2.8 (Figure S27d, Supporting Information), suggesting the predominance of a two‐electron transfer route in the ORR process. Notably, the Fe/Fe_3_C/C (0.61 V vs RHE and −4.08 mA∙cm^−2^) exhibits an apparent enhancement of catalytic activity for ORR as compared to control catalyst NC. Importantly, it displays the predominance of a four‐electron transfer route in the ORR process (*n* = 3.78) (Figure S27b, Supporting Information), indicating the superior ORR activity of Fe/Fe_3_C. Notably, as compared to NC, the half‐wave potential and limiting current density of Fe_0.5_‐N‐C@TABOH (0.69 V vs RHE and −4.65 mA∙cm^−2^) are remarkably improved after the fabrication of FeN*_x_* sites into NC, with the half‐wave potential positively shifted by 27 V. This improvement originates from the fabrication of FeN*_x_* sites into NC. In addition, the Fe_0.5_‐N‐C@TABOH shows superior ORR activity to Fe/Fe_3_C/C, with the half‐wave potential higher than that of Fe/Fe_3_C/C by 8 V. Crucially, Fe_4_‐N‐C@TABOH (0.71 V vs RHE and −5.23 mA∙cm^−2^), which combines FeN*_x_* and Fe/Fe_3_C sites with NC, displays the most excellent ORR activity among those of the control catalysts Fe_0.5_‐N‐C@TABOH, Fe/Fe_3_C/C and NC. The comparison of ORR activity over Fe_4_‐N‐C@TABOH and Fe_0.5_‐N‐C@TABOH indicates the removal of Fe/Fe_3_C nanocrystals (Fe_0.5_‐N‐C@TABOH) decreases ORR activity, confirming the positive effect of Fe/Fe_3_C sites on improving ORR activity. The above finding indicates that FeN*_x_* sites are highly active for ORR and their ORR activity can be further improved by introducing Fe/Fe_3_C nanoparticles into FeN*_x_* sites through synergistic effect between FeN*_x_* and Fe/Fe_3_C sites. To further evaluate the ORR activity of different active sites, turnover frequency (TOF) values at 0.6 V of these catalysts were calculated (Figure S28, Supporting Information). The TOF value of Fe_0.5_‐N‐C@TABOH (the case of FeN*_x_* site) is 0.53 e·s^−1^, which is remarkably higher than that of Fe/Fe_3_C/C (Fe/Fe_3_C sites), and even higher than Fe_4_‐N‐C@TABOH (combined FeN*_x_* and Fe/Fe_3_C sites). Overall, FeN*_x_* sites have high intrinsic activity for ORR and the synergistic effect between FeN*_x_* and Fe/Fe_3_C sites induced the excellent ORR performance.

#### 2.4.4 DFT Analysis of Different Fe‐Nx Coordinations

To delve deeper into the mechanism driving the exceptional ORR performance of Fe_4_‐N‐C@TABOH, DFT calculations were employed to evaluate the free energy of each intermediate reaction step. A model (M‐FeN_4_) depicting FeN_4_ surrounded by 10 carbons and one graphitic N (where the supercell consists of 64 carbon atoms) was devised for microporous Fe_4_‐N‐C (Figure 8a), while for Fe_4_‐N‐C@TABOH, characterized by a blend of micropores and mesopores, the model was adjusted by introducing two‐edged C sites representing the mesopores or defects (termed as D‐FeN_4_, as shown in Figure 8c). Similar models were crafted for FeN_4_ structure by Wang et al.^[^ ^36^ ^]^ Correspondingly, models M‐FeN_5_ (Figure 8b) and D‐FeN_5_ (Figure 8d) were established for both catalysts. The optimized structures of ^*^O, ^*^OH, and ^*^OOH adsorbed on these models are depicted in (Figure 8a–d). Figure 8e,f, summarizes the trends of calculated free energy of intermediate structures at the initial state of 0 V and the equilibrium state of 1.23 V. It is notable that the free energy decreases successively up to ^*^OH for all four coordination structures at 0 V, indicating the propensity for the ORR process to occur on these structures. Particularly, the desorption of ^*^OH emerges as the rate‐determining step for all models. The D‐FeN_5_ (0.12 eV) model exhibits the lowest energy barrier among the FeN_5_ (0.50 eV), D‐FeN_4_ (0.79 eV), and FeN_4_ (1.09 eV), emphasizing the facilitative role of the structure tailoring and microenvironment optimization by the TABOH in reducing O_2_ to H_2_O.

As shown in Figure S29 (Supporting Information), for D‐FeN_5_, a significant redistribution of electrons can be observed, affirming the role of TABOH in electronic modulation. This will further optimize the electron distribution on Fe centers, forming an electron‐rich region around Fe atoms, which is consistent with the results of XANES and Mössbauer spectroscopy analysis. This could significantly boost the ORR catalytic activity of D‐FeN_5_. Moreover, the projected density of states (PDOS) in d‐band was used to evaluate the optimization effect of TABOH on electron microenvironment. As shown in Figure S30 (Supporting Information), the d‐band center of D‐FeN_5_ (−2.27 eV) is shifted to a lower energy level compared to M‐FeN_4_ (−1.66 eV). According to the d‐band center theory, the occupied states are closer to the Fermi energy level, resulting in a higher binding energy of O_2_.^[^ ^37^ ^]^ Therefore, D‐FeN_5_ has a low binding energy with O_2_, which facilitates O_2_ adsorption.

In summary, in tandem with the EXAFS and ^57^Fe Mössbauer spectra analysis, it can be inferred that apart from expediting the synthesis process and increasing yield of the Zn/Fe‐ZIF‐H precursor, TABOH also induces a hierarchical porous structure and fosters the formation of more and higher intrinsic active FeN_4_ and FeN_5_ ORR sites through its unique and irreplaceable electronic modulation function. And the remarkable ORR performance of Fe_4_‐N‐C@TABOH can be attributed to several factors: 1) Its substantial mesoporosity and extensive external surface area augment the accessibility of ORR active sites and enhance their efficiency of utilization. 2) The significant *V*_meso_, along with the hierarchically porous structure and high graphitic N in Fe_4_‐N‐C@TABOH, facilitates rapid mass/electron transfer and reduces internal resistance, thereby greatly enhancing MFC output power density through the external circuit. 3) Fe_4_‐N‐C@TABOH harbors an increased abundance of higher intrinsic active FeN_4_ and FeN_5_ sites, contributing to its superior ORR activity. 4) The synergistic effect between FeN*_x_* and Fe/Fe_3_C sites induced excellent ORR performance. 5) The proximity of metal iron atoms near the FeN*_x_* active site results in an elevation of the highest occupied orbital of Fe atom within the FeN*_x_* sites, facilitating its overlap with the p orbital of the oxygen molecule. This enhances oxygen adsorption and thereby promotes the ORR process.

## 3 Conclusion

Highly active hierarchical porous Fe*_x_*‐N‐C@TABOH ORR catalysts were successfully fabricated by a facile and rapid strategy using TABOH as a structural template and electronic mediator at room temperature within a very short time of 16 min, which greatly improved the preparation efficiency and increased the yield. The obtained Fe*_x_*‐N‐C@TABOH demonstrated superior ORR performance, which can be attributed to its high mesoporous volume, hierarchical porous structure, synergistic effect between FeN*_x_* and Fe/Fe_3_C sites and uniform distribution of highly intrinsically active FeN*_x_* sites (D1 and D3). Crucially, the average electron transfer number (*n*) of Fe_4_‐N‐C@TABOH measured at 3.84 validates the predominance of a four‐electron transfer route in the ORR process. MFC assembled with Fe_4_‐N‐C@TABOH showed superior electrical performance compared to other Fe*_x_*‐N‐C@TABOH catalysts, offering an exceptionally high MFC power density of 830.1 ±5.2 mW·m^−2^, nearing that of the Pt/C catalyst. Moreover, the output voltage plots of MFCs incorporating Fe_4_‐N‐C@TABOH exhibited remarkable stability, with no discernible downward trend observed over the 75‐day operational period. Both the experimental results theoretical analysis further elucidated that beyond expediting synthesis and increasing yield of the Zn/Fe‐ZIF‐H precursor, the TABOH template induces a hierarchical porous structure and fosters the formation of more and higher intrinsic active FeN*_x_* moieties in Fe*_x_*‐N‐C@TABOH catalysts. This strategy holds new idea for diverse applications of catalysts based on ZIF precursors.

## Conflict of Interest

The authors declare no conflict of interest.

## Author Contributions

B.J. wrote the original draft (Lead) and review and performed editing (Lead); N.J. and J.L. performed methodology (Supporting); Y.C. performed investigation (Supporting) and wrote the original draft (Supporting); H.W. wrote the review and performed editing (Supporting); G.Z. performed formal analysis (Supporting)Y.Z. performed investigation (Supporting).

## Supporting information

Supporting Information

## Data Availability

Data sharing is not applicable to this article as no new data were created or analyzed in this study.
