# Supplementary material for: Rapid Synthesis and Microenvironment Optimization of Hierarchical Porous Fe─N─C Catalysts for Enhanced ORR in Microbial Fuel Cells
Source: Adv Sci (Weinh). 2024 Jun 17;11(31):2402610. doi: 10.1002/advs.202402610 (PMC11336980; doi:10.1002/advs.202402610)
Supplement: Supplementary file 1 — Supporting Information [file ADVS-11-2402610-s001.docx]

**Rapid Synthesis and Microenvironment Optimization of Hierarchical Porous Fe-N-C Catalysts for Enhanced ORR in Microbial Fuel Cells**

Bolong Jiang^a,b,c,^*, Nan Jiang^a^, Yanyan Cui^a,b^, Huan Wang^c^, Geng Zhang^c^, Jiayou Li^a,b^, Yuhan Zhang^a,b^.

^a^Innovation Institute for Sustainable Maritime Architecture Research and Technology,

Qingdao University of Technology, Qingdao 266000, Shandong, China.

^b^Institute of Environmental and Municipal Engineering, Qingdao University of Technology, Qingdao 266000, Shandong, China.

^c^College of Chemistry and Chemical Engineering, Northeast Petroleum University, Daqing 163318, Heilongjiang, China.

^*^Corresponding author Tel./Fax: +86 0532 85071522

Email: jiangbolong@qut.edu.cn

**1 Experimental**

**1.1 Synthesis of the catalysts**

**Synthesis of Zn/Fe*_x_*-ZIF-TABOH and Fe*_x_*-N-C@TABOH:** A facile and fast strategy to synthesis hierarchical porous Zn/Fe-ZIF-TABOH precursors were proposed and used to synthetize Fe*_x_*-N-C@TABOH catalysts. Typically, 1.9704 g of 2-methylimidazole and 0.66 g of zinc acetate (C_4_H_6_O_4_Zn) and FeSO_4_∙7H_2_O (with Fe^2+^/(Fe^2+^+Zn^2+^) molar ratio of 0.5%, 1%, 2%, 4% and 8% were dissolved in 5 mL CH_3_OH and fully stirred at room temperature. Then, 0.2 mL of tetrabutylammonium hydroxide (TABOH, Fig.S1) was added into the solution under stirring and stirred for 16 min. The suspension was then filtered, washed thoroughly and dried to yield Zn/Fe*_x_*-ZIF-TABOH powder. The powder was pyrolyzed at 900 ℃ for 2 h in a flowing Ar_2_ (3 °C·min^-1^). The obtained solids were immersed in 0.1 M HCl for 24 h and donated as Fe*_x_*-N-C@TABOH, where *x* is the Fe^2+^/(Fe^2+^+Zn^2+^) molar ratio and is expressed in %.

**Synthesis of Zn/Fe_4_-ZIF-N and Fe_4_-N-C:** The synthesis of Zn/Fe_4_-ZIF-N was basically identical to the preparation of Zn/Fe_4_-ZIF-TABOH, but TABOH was not added and reaction time was extended to 24 h. And the Fe_4_-N-C was obtained from Zn/Fe_4_-ZIF-N through the same method of Fe_4_-N-C@TABOH.

**Synthesis of Fe/Fe_3_C/C and Nitrogen-doped carbon (NC) catalyst**: Fe/Fe_3_C/C catalyst was prepared by one-step hydrothermal method, in which terephthalic acid (PTA) and Fe(NO_3_)_3_∙9H_2_O were dissolved in 10 mL N, N-dimethylformamide (DMF). The resulting solution was stirred at room temperature for 30 min and transferred to 50 mL Teflon autoclave and heated at 120 ℃ for 15 h. The suspension was then filtered and washed thoroughly and dried to yield catalyst precursor. The catalyst precursor was treated with the same method as Zn/Fe*_x_*-ZIF-TABOH and the as prepared catalyst with Fe content of 4.566 wt.% is named as Fe/Fe_3_C/C.

Nitrogen-doped carbon (NC) catalyst was prepared by the same method of Fe_4_-N-C@TABOH, except that Fe species is not added to ensure that the resulting catalyst does not contain any active Fe sites.

**1.2 Electrode fabrication**

Fabrication of cathode: firstly, to make the carbon-based layer, 50 mg carbon black (Vulcan xc-72, USA) and 600 μL PTFE emulsion (60 wt%, Shanghai Hesen Electric Co., Ltd., China) are mixed thoroughly and evenly coated on a 4×4 cm carbon fiber cloth (30% waterproof treatment, American ETEK), and treated at 370 °C for 20 min to form the carbon layer. To make gas diffusion layer, the surface of carbon layer was then brushed with 60% PTFE emulsion and dried in the air, and then heated at 370 °C for 20 min. After cooling to room temperature, the operation of brushing, air-drying, heating and cooling was repeated for three times, which constructed the stable diffusion layer. The obtained carbon cloth was tailored to a wafer with a diameter of 4 cm. Finally, the catalytic layer (5 mg·cm^-2^) was coated on the opposite side of diffusion layer and dried in the air for 24 h. the catalytic layer was made of 60 mg catalyst with 50 μL deionized water, 400 μL Nafion emulsion (5 %, Sigma-Aldrich co., LTD, China), and 200 μL propanol (Alladdin, Biochemical Technology Co., Ltd., Shanghai, China). For comparison, Pt/C (20 wt% Pt, E-TEK, USA) was also coated by the same method.

Treatment of anode electrode: The anodes of all reactors were employed carbon fiber brush. Carbon brushes with a diameter of 3 mm and a length of 2.5 cm were prepared to meet the volume of the electrolytic cell. Before using, the brush was soaked in acetone solution for 24 h to remove contaminants from the brush preparation and washed by several times with distilled water. Finally, it was heat treated at 470 °C for 30 min and cooled at room temperature.

**1.3 MFC setup and operation**

The cube-shaped, single chamber MFC (Volume of 28 mL) was constructed. The graphite brush anode (diameter of 3.0 cm and length of 2.5 cm) was placed horizontally in the middle of the cylindrical chamber. The cathode was fixed at the other side of MFC, with the catalyst layer facing the electrolyte. The cathode of MFC was carbon cloth with a catalytic layer of Fe*_x_*_%_-N-C@TABOH. All MFCs were inoculated with 20% wastewater collected from a sewage treatment plant and 80% nutrient medium contained glucose (1 g·L^-1^), phosphate buffer solution (0.05 M), vitamins (5 mL·L^-1^) and minerals solution (12.5 mL·L^-1^). The MFCs were operated in a 30^o^C incubator with external resistance of 1000 Ω and each experiment was performed 4 times in parallel. The nutrient medium was replaced when the voltage output decreased below 50 mV.

**1.4 Structural characterization**

The surface area and pore volume were measured using the Brunauer-Emmett-Teller (BET, Micromeritics, NOVA 2000e：Surface Area & Pore Size Analyzer). The morphology was analyzed using scanning electron microscopy (SEM, JSM-5600LV). The Transmission electron microscopy (TEM) analysis was performed using a TEM (HT770TEM). The X-ray diffraction (XRD) measurements were recorded on an X-ray diffractometer (a D/max-2200PC-X-ray diffractometer, 40 kV, 30 mA) using a Cu Kα radiation source in the scan range from 10° to 80° at a rate of 10°·min^−1^. The XPS photoelectron spectroscopy was conducted on ESCALAB MKII spectrometer with monochromatic Mg *K*α source (E=1253.6 eV) at a constant pass energy of 40 eV. The recorded binding energies were referenced against the C1s contamination line at 284.8 eV. Fourier transform infrared (FT-IR) spectra of the samples in the form of KBr pellets were collected on an FT-IR spectrometer (Bruker Tensor 27) with a resolution of 4 cm^-1^. Raman spectrum was obtained by using a 532 nm laser on a Raman spectrometer (LabRAM HR Evolution, HORIBA Scientific, Japan) to determine the degree of graphitization of synthesized carbon materials. Metal loadings of the samples were determined by inductively coupled plasma atomic emission spectrometry (ICP-AES) on an Optima 7300 DV. Elemental analysis was performed on a Vario Macro elemental analyzer. Raman spectra were collected on an XploRA PLUS spectrometer with a 532 nm excitation laser. The X-ray absorption fine structure (XAFS) spectroscopy at Fe K-edge was performed at the BL08B2* of SPring-8 (8 GeV, 100 mA), Japan, in which, the X-ray beam was mono-chromatized with water-cooled Si (111) double-crystal monochromator and focused with two Rh coated focusing mirrors with the beam size of 2.0 mm in the horizontal direction and 0.5 mm in the vertical direction around sample position, to obtain XAFS spectra both in near and extended edge. Fe foil, Fe_2_O_3_, FePc and FeS_2_ samples were used as references. The obtained XAFS data was processed in Athena (version 0.9.26) for background, pre-edge line and post-edge line calibrations. Then Fourier transformed fitting was carried out in Artemis (version 0.9.26). The k^3^ weighting, k-range of 3 - 14 Å^-1^ and R range of 1 - ~3 Å were used for the fitting of Fe foil; k-range of 3 - 12 Å^-1^ and R range of 1 - ~3 Å were used for the fitting of samples. The four parameters, coordination number, bond length, Debye-Waller factor and E_0_ shift (CN, R, ΔE_0_) were fitted without anyone was fixed, the σ^2^ was set. For Wavelet Transform analysis, the χ(k) exported from Athena was imported into the Hama Fortran code. The parameters were listed as follow: R range, 1 - 4 Å, k range, 0 - 15 Å^-1^ for samples; k weight, 3; and Morlet function with κ=10, σ=1 was used as the mother wavelet to provide the overall distribution. The ^57^Fe Mössbauer spectra of compounds 1 and 2 were recorded on an SEE Co W304 Mössbauer spectrometer, using a 57Co/Rh source in transmission geometry. The data were fitted by using the MossWinn 4.0 software.

**1.5 Electrochemical test**

All tests were conducted in a three-electrode system equipped to an electrochemical workstation (CHI760E) at room temperature (25 ± 1 °C). The catalyst ink was prepared with 4 mg of catalyst, 400 μL ethanol, 100 μL water and 25 μL 5 wt% Nafion ultrasound for 40 min and coated evenly on the surface of RDE for further tests. The measurements were carried out in 0.1 M PBS (Na_2_HPO_4_, 9.15 g·L^-1^; NaH_2_PO_4_, 4.90 g·L^-1^; NH_4_Cl, 0.62 g·L^-1^; KCl, 0.26 g·L^-1^; PH=7.4) buffer solution with saturated O_2_. Platinum plate and saturated saturated RHE were used as counter electrode and reference electrode respectively. Cyclic Voltammetry (CV) is widely used to test the electrochemically active area (ECSA) of fuel cell electrodes. CVs were recorded from 0.90 to 1.0 V vs RHE at a scan rate of 100, 90, 80, 70, 60, 50, 40, 30, 20, 10 mV s^−1^ to determine the double layer capacitance (C_dl_) from the slope of the linear fit of the current vs scan rate. The electrochemical surface area (ECSA) is calculated by the following equation^[1]^:

$ECSA=(\frac{C_{dl}}{C_{S}})A$ (1)

where A is the geometric area of electrode and Cs is the specific capacitance of the sample corresponding to a smooth surface under the same conditions. The specific capacitances have been measured for a variety of metal electrodes in acidic and alkaline solutions and typical values reported range between C_s_ = 0.015−0.110 mF cm^−2^ in H_2_SO_4_ and C_s_ = 0.022−0.130 mF cm^−2^ in NaOH and KOH solutions. In general, the specific capacitance C_s_ = 0.035 mF cm^−2^ in 1 M H_2_SO_4_ and C_s_ = 0.040 mF cm^−2^ in 1 M NaOH based on typical reported values^[1]^. Considering that MFC works under neutral conditions, based on C_s_ values under acidic and alkaline conditions, we take 0.038 mF·cm^-2^ when calculating ECSA.

Cyclic voltammetry (CVs) scanning rates of 50 mV·s^−1^ were recorded in saturated O_2_ solutions. The linear sweep voltammetry (LSV) of Fe@N-C was conducted by RDE under 1600 rpm in 0.1 M PBS solution with saturated O_2_. The Nyquist curves of Pt/C and Fe*_x_*_%_-N-C@TABOH catalysts for EIS analysis were measured by adding alternating current (AC) with frequency of 0.1~10^5^ Hz between anode and cathode. For rotating disk electrode (RDE) test, the polarization curves were collected at disk rotation rates of 400～2000 rpm. To the calculate the electron transmitting number (n) of ORR, the LSV curves were analyzed on the basis of Koutecky–Levich (K-L) equations,

$\frac{1}{J}=\frac{1}{J_{L}}+\frac{1}{J_{K}}=\frac{1}{0.62nFC_{o}{{(D}_{o})}^{2/3}v^{(-1/6)}\omega^{1/2}}+\frac{1}{J_{k}}$ (2)

$J_{K}=\frac{1}{nkFC_{0}}$ (3)

The J, J_L_ and J_k_ are the measured, diffusion-limiting and kinetic limiting current densities in mA·cm^-2^, respectively. The F (C·mol^-1^) is refer to the Faraday constant (F=96485), ω (rad·s^-1^) represents the angular velocity of the disk; C_o_ (mol·cm^-3^) and D_0_ (cm^2^·s^-1^) are the bulk concentration and the diffusion coefficient of O_2_ in 0.1 M neutral phosphate buffer solution in, respectively, and υ (cm^2^·s^-1^) is the kinematic viscosity of the electrolyte.

The kinetic current was calculated from the mass-transport correction of RDE by the following equation:

$J_{K}=\frac{J_{L}\times J}{J_{L}- J}$ (4)

The electron transfer number ($n$) and the yield of hydrogen peroxide (H_2_O_2_, %) were determined through rotating ring-disk electrode (RRDE) measurement (at 1600 rpm) and were calculated by the equations as follows:

$n=4\times\frac{I_{d}}{I_{d}+\frac{I_{r}}{N}}$ (5)

$H_{2}O_{2}(\%)=\frac{200I_{r}}{N\cdot I_{d}+I_{r}}$ (6)

where $N$ (= 0.37) is the current collection efficiency of the Pt ring in RRDE, $I_{r}$ and $I_{d}$ present the and ring and disk current, respectively.

The turnover frequency (TOF) is calculated by normalizing the kinetic current density based on the following equations:

$TOF=\frac{j_{K}N_{e}M_{Fe}}{C_{cat}N_{A}W_{Fe}}$ (7)

where N_e_ represents the electron number of per Coulomb (6.24×10^18^ e C^-1^), M_Fe_ represents the molar mass of Fe (55.845 g mol^-1^), N_A_ represents the Avogadro constant (6.022×10^23^ mol^-1^), C_cat_ represents the catalyst loading (0.2 mg cm^-2^), W_Fe_ represents the weight percentage of Fe measured by ICP-OES.

**1.6 Theoretical calculation**

The ZIF precursors (i.e. the chelates composed of Zn^2+^ and 2-methylimidazole), methanol and TMAH template were selected as the simulation objects and described using a coarse-grained model, thus they could be regarded as the combination of the spring and beads^1^. Four beads were defined as follow: C beads (CH_4_), O beads (NOH), Z beads (the ZIF precursors) and M beads (methanol). The topology of TMAH template was expressed as the O [C][C][C][C]. Therefore, the species in the system were Z, M and O [C][C][C][C] with their relative amount was 1:4:1.

After optimizing the structures of the four beads by the classical Forcite tool, the interaction parameters were calculated by the Blends module, as shown in Table S1. The repulsion parameter *α_ij_* between bead *i* and bead *j* could be derived from the equation: *α_ij_* = *α_ii_* + 3.27*χ_ij_* (where *α_ii_* is the repulsive parameter among the same beads and *χ_ij_* is the Flory-Huggins parameter)^2^. In this work, *α_ii_* = 25 basing on the compressibility of pure liquid^3^. The size of the simulated box was set as 32 × 32 × 32 nm and the grid spacing was 1.0 nm. The bond length was fixed at 1.1543 to ensure isotropy of all grid restricted operators. To simulate the stirring operation during the synthesis process, a uniform steady shear was introduced to the simulation system after 0.25 ms^4^ and the shear rate was set as 0.00075 ns^-1^. The number of steps was 20000 (the total simulation time was 1.0 ms) and the time step was kept at the default 50.0 ns. The bead diffusion coefficient was set as 1.0e-7 cm^2^·s^-1^.

**Table S** Flory-Huggins interaction parameter *χ* between beads

| Bead | C | O | Z | M |
| --- | --- | --- | --- | --- |
| C | 0.0 | 0.10245 | 2.7870 | 0.019975 |
| O | 0.10245 | 0.0 | 4.4276 | 0.10916 |
| Z | 2.7870 | 4.4276 | 0.0 | 2.7048 |
| M | 0.019976 | 0.10916 | 2.7048 | 0.0 |

The free energy change in Gibbs (∆G) of ΔG ORR intermediate with respect to liquid H_2_O was calculated using the following equation by Nøskov et al.:
 ΔG = ΔE +ΔZPE-TΔS (8)

Where, ΔE, ΔZPE and ΔS denote the DFT-calculated ground state energy, zero-point energy, and entropy corrections, respectively.

Five electron reaction pathways were considered, as shown in equ (9)-(12):

O_2_+e^-^+H_2_O→OOH*+OH^-^ (9)

OOH*+e^-^→O*+OH^-^ (10)

O*+e^-^+H_2_O→OH*+OH^-^ (11)

OH*+e^-^→*+OH^-^ (12)

The d-band center proposed by Nurskov and co-workers ^[2]^ is a semi-quantitative descriptor to describe the trend of reactivity of transition metals (TM), which is defined the d-band center (ε_d_) relative to the Fermi level (EF). A transition metal with a low ε_d_ value relative to the Fermi level, shows a weak adsorption for a given adsorbate. The d-band center (ε_d_) values were calculated as follows:

$\varepsilon_{d}=\frac{\int_{-\infty}^{+\infty} x\rho\left( x \right)dx}{\int_{-\infty}^{+\infty} \rho\left( x \right)dx}$ (13)

where ρ(x) is the projected density of states (PDOS) with respect to a Fe atom on the models.

**2 Figures**


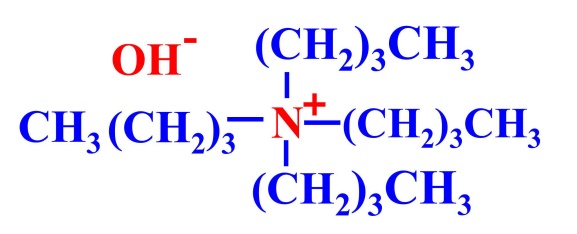


Fig.S1 Structure of tetrabutylammonium hydroxide template

Fig.S2 XRD images of Fe*_x_*-N-C@TABOH

Fig.S3 N_2_ adsorption-desorption isotherms and pore size distributions of Fe*_x_*-N-C@TABOH

Fig.S4 XPS survey of Fe*_x_*-N-C@TABOH

Fig.S5 XPS spectra of Fe*_x_*-N-C@TABOH (N 1s)

Fig.S6 XPS spectra of Fe*_x_*-N-C@TABOH (Fe 2p).

Fig.S7 XPS spectra of Fe*_x_*-N-C@TABOH (C 1s).

Fig.S8 XPS spectra of Fe*_x_*-N-C@TABOH (O 1s).

Fig.S9 Raman spectrum of Fe_4_-N-C@TABOH and Fe_4_-N-C

Fig.S10 CV curves of Fe*_x_*-N-C@TABOH in O_2_ saturated 0.1 M neutral PBS solution.

Fig. S11 CV curves (a-e) and calculated Cdl results (f) of Fe*_x_*-N-C@TABOH and Fe_4_-N-C recorded in 0.1 M PBS.

Fig. S12 ECSA-normalized LSV of Fe*_x_*-N-C@TABOH and Fe_4_-N-C catalysts

Fig.S13 Electron transfer number (n) and H_2_O_2_ yield (a), TOF of Fe_4_-N-C@TABOH.

Fig. S14 LSV plots of 20%Pt/C before and after 20000 potential cycles.


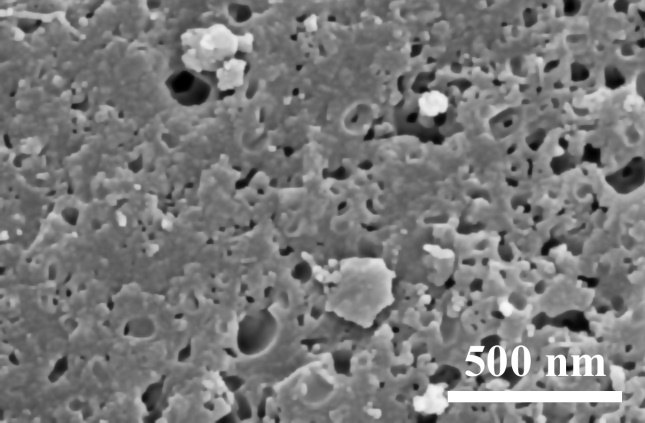


Fig.S15 XRD curves and SEM image of Fe_4_-N-C@TABOH after stability test

Fig.S16 Cathode and anode polarization curves of Fe_4_-N-C@TABOH, Fe_4_-N-C and 20% Pt.


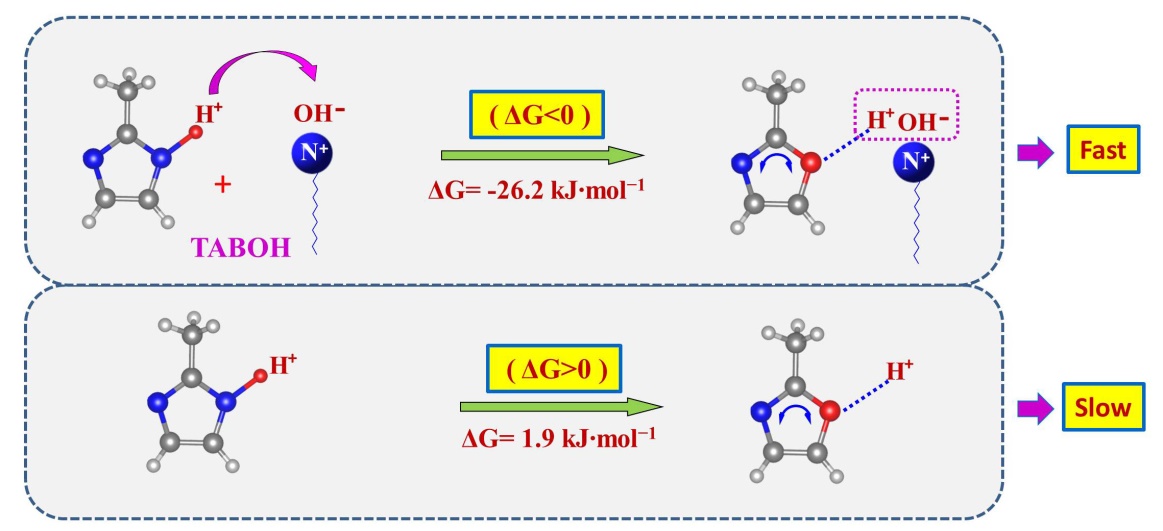


Fig.S17 Breaking energy of H-N bond of 2-MI with TABOH and without TABOH.


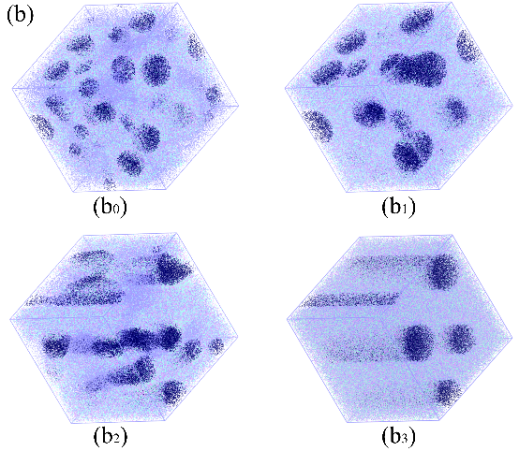


Fig. S18 Time evolution of the order parameter *P* during the mesophase formation process (a); Snapshots of the system (b) at 0.0 min (b_0_), 0.25 min (b_1_), 0.5 min (b_2_), 1.0 min (b_3_).

Fig.S19 XRD analysis of Zn/Fe_4_-ZIF-H samples

Fig. S20 FT-IR spectra of Zn/Fe_4_-ZIF-H, Zn/Fe_4_-ZIF-N precursors and Zn-ZIF-H, Zn-ZIF-N samples.

Fig.S21 N_2_ adsorption-desorption isotherms and BJH pore size distributions of Zn/Fe_4_-ZIF-N and Zn/Fe_4_-ZIF-H


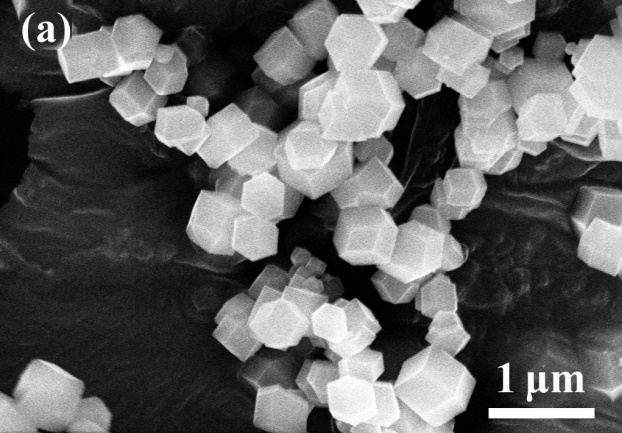

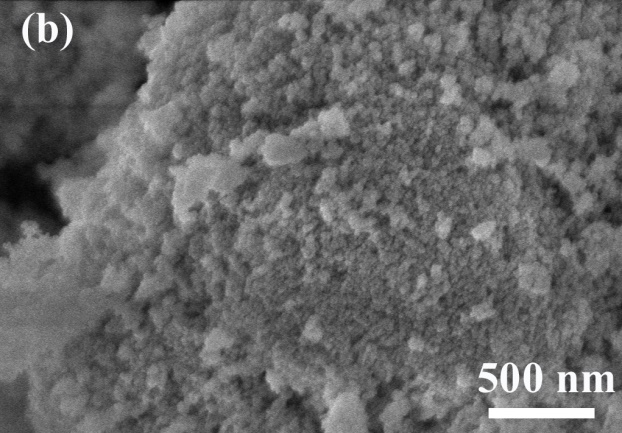


Fig.S22 SEM images of Zn/Fe_4_-ZIF-N and Zn/Fe_4_-ZIF-H

Fig.S23 WT contour plots of Fe foil (a), FePc (b), Fe_4_-N-C@TABOH (c) and Fe_4_-N-C (d).

Fig.S24 XRD patterns of Fe/Fe_3_C/C, Fe_0.5_-N-C@TABOH and NC catalysts.


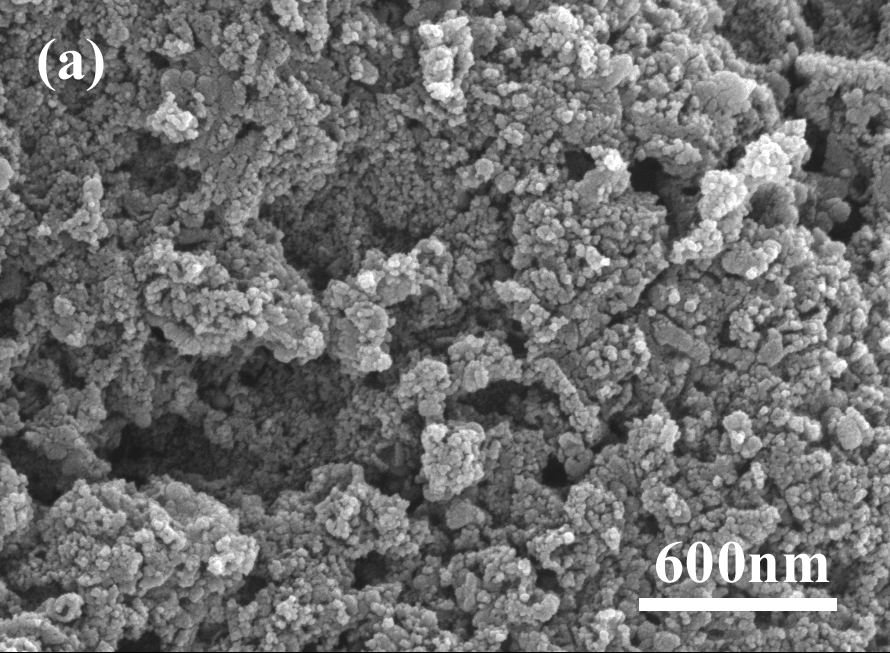

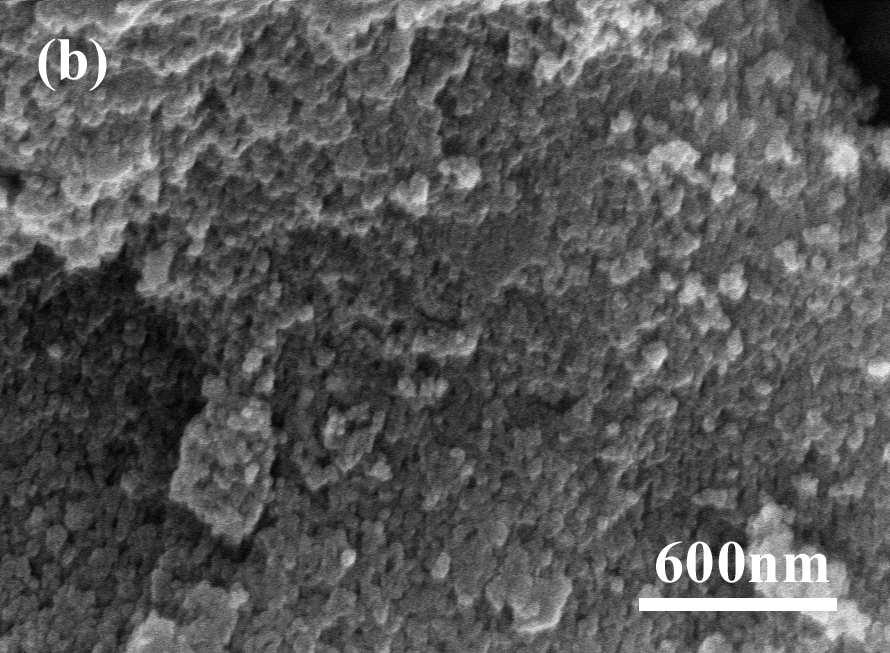

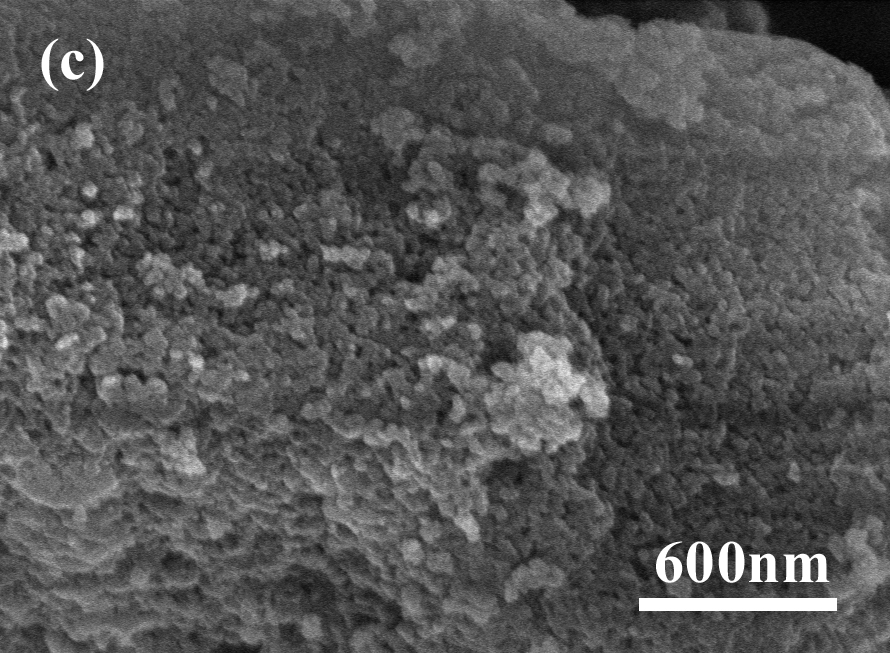


Fig.S25 SEM images of Fe/Fe_3_C/C (a), Fe_0.5_-N-C@TABOH, (b) and NC (c) catalysts.


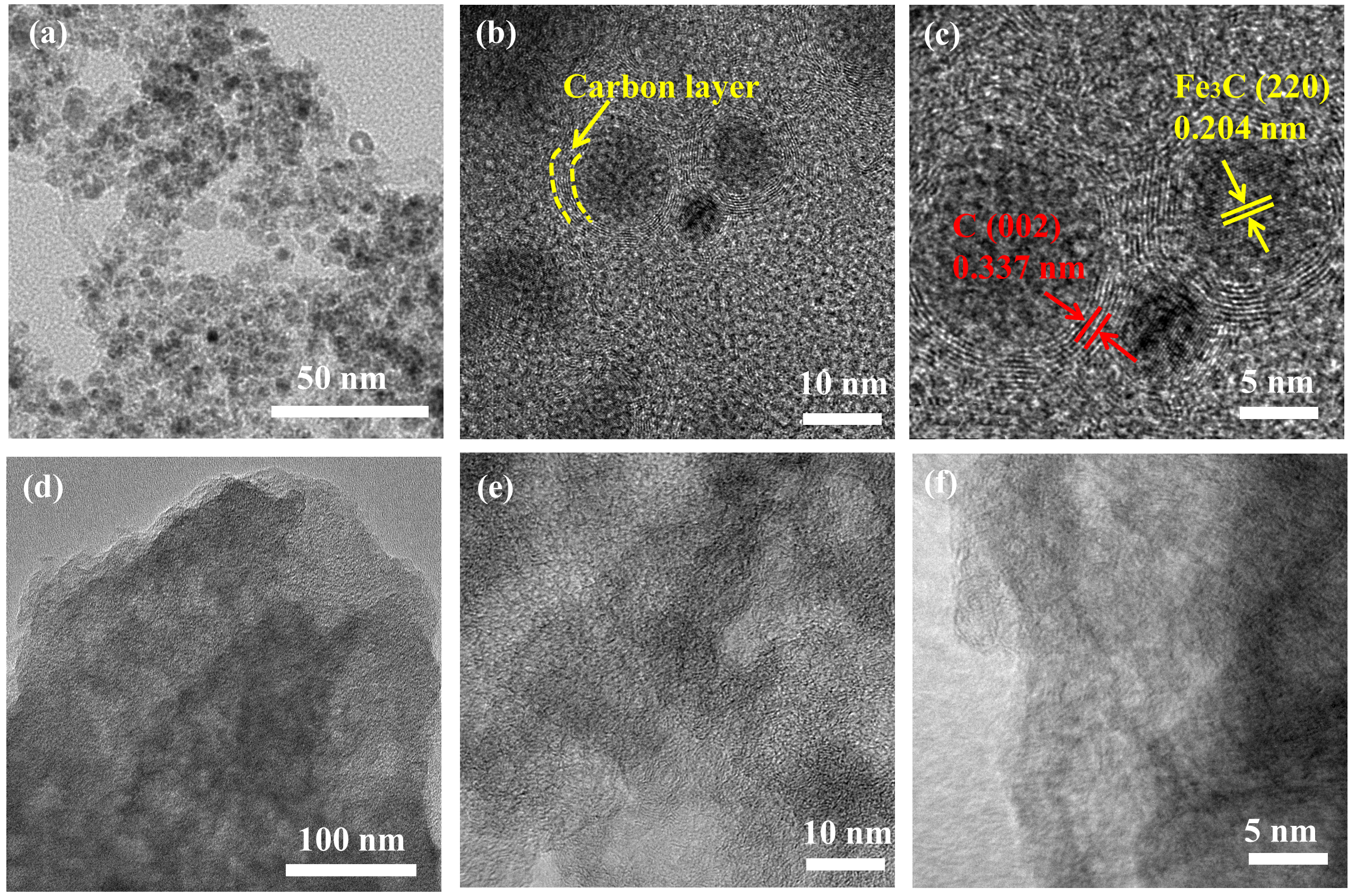


Fig.S26 TEM images of Fe/Fe_3_C/C (a-c) and Fe_0.5_-N-C@TABOH (d-f) catalysts.


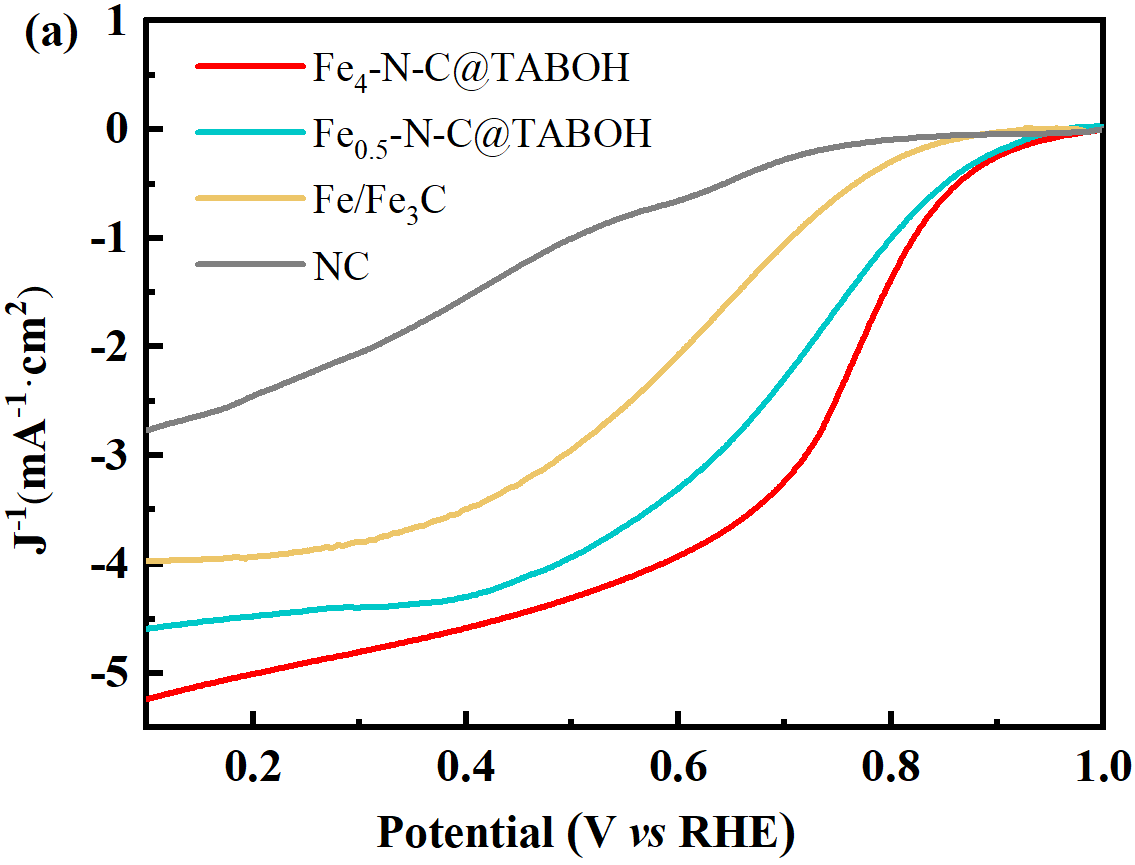

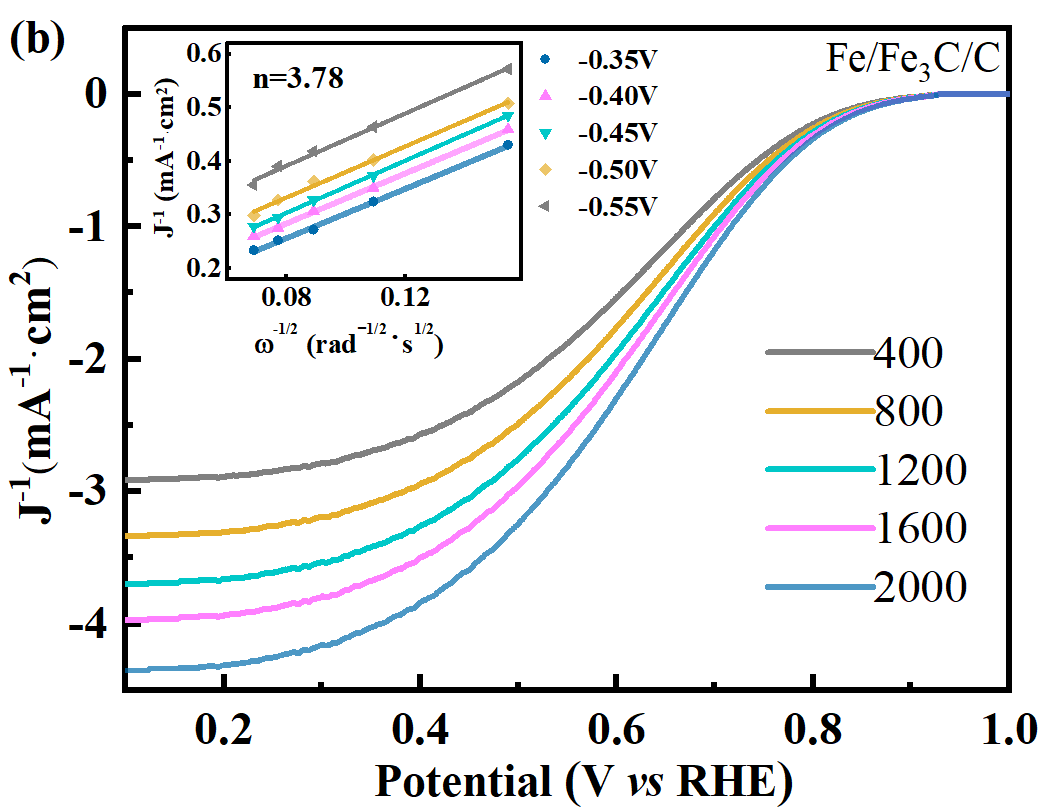

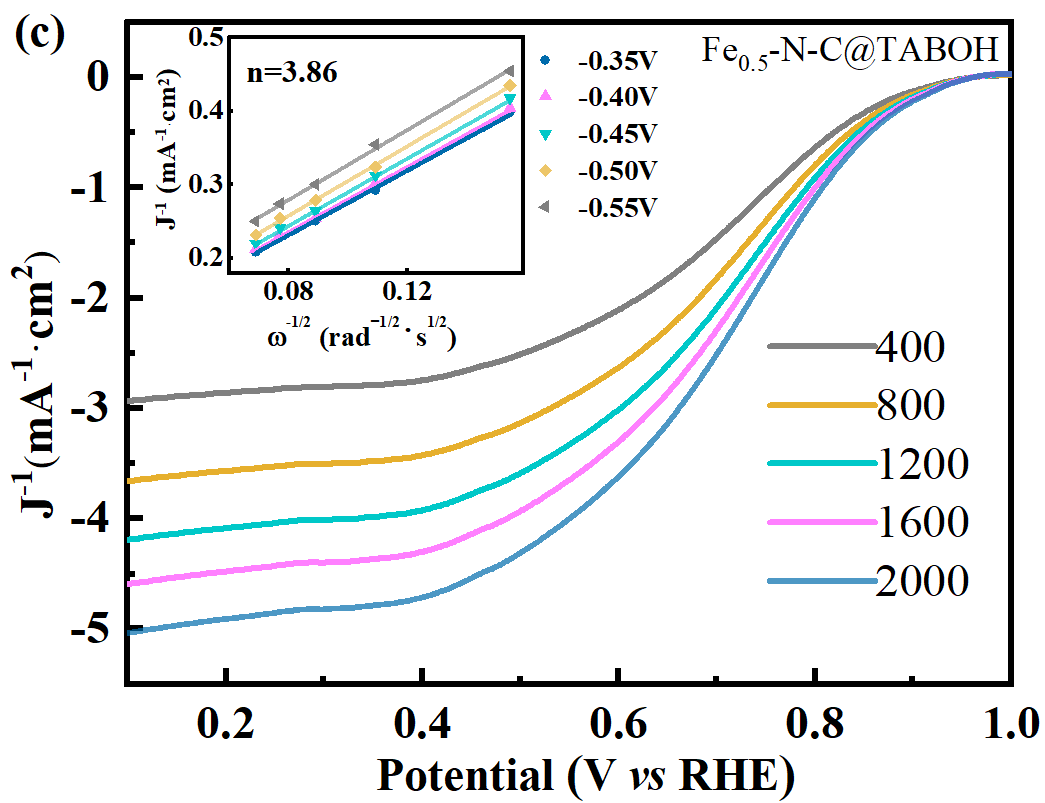

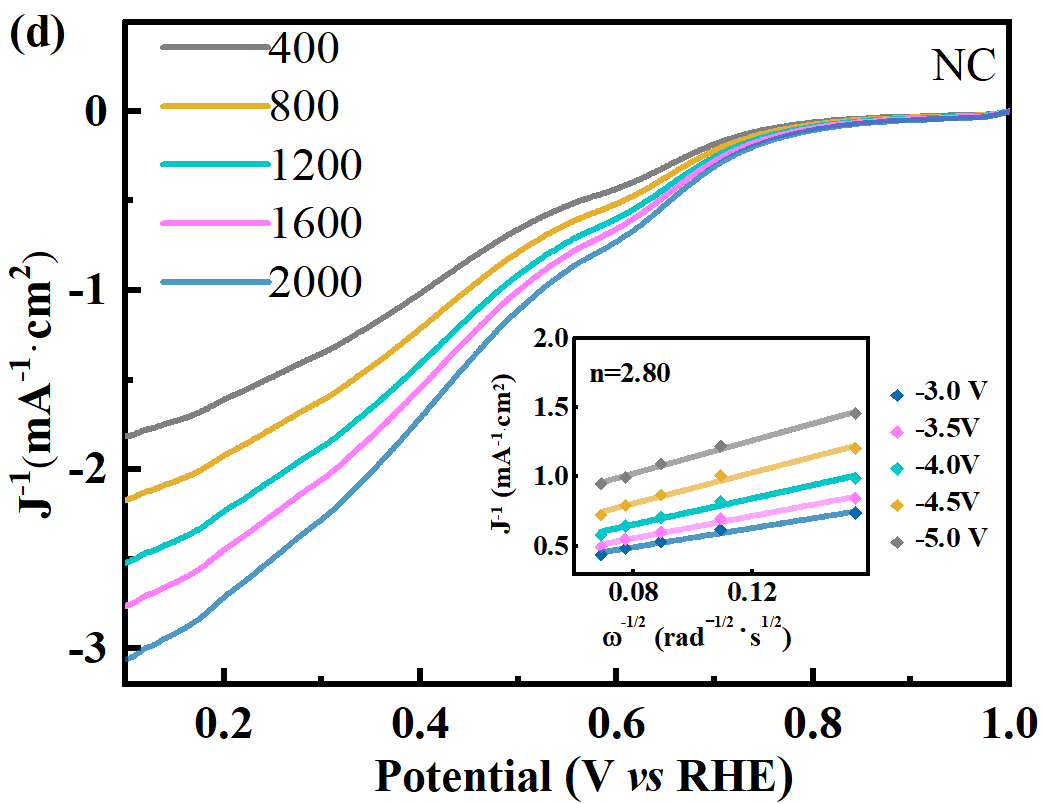


Fig.S27 LSV and Koutecky-Levich (inset) plots for Fe/Fe_3_C/C, Fe_4_-N-C@TABOH and NC.

Fig.S28 *E*_1/2_ and TOF of Fe/Fe_3_C/C, Fe_0.5_-N-C@TABOH and NC in 0.1 M PBS electrolyte.


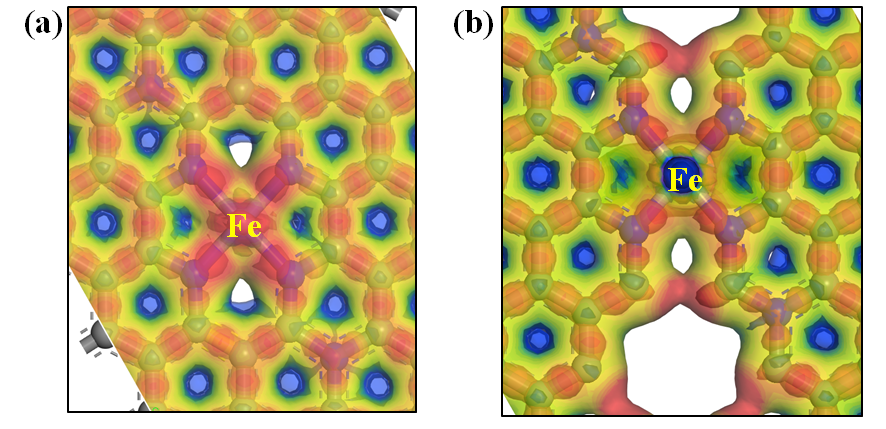


Fig.S29 The charge density difference images of M-FeN_4_ (a) and D-FeN_5_ (b).

Fig.S30 Density of state plots and the d-band centers of M-FeN_4_ and D-FeN_5_.

**3 Tables**

Table S1 BET results of Fe*_x_*-N-C@TABOH and Fe_4_-N-C catalysts

| Samples | S_BET_ (m^2^·g^-1^) | S_external_ (m^2^·g^-1^) | V_p_  (cm^3^·g^-1^) | V_micro_  (cm^3^·g^-1^) | V_meso_  (cm^3^·g^-1^) |
| --- | --- | --- | --- | --- | --- |
| Fe_1_-N-C@TABOH | 548 | 199 | 0.37 | 0.22 | 0.15 |
| Fe_2_-N-C@TABOH | 530 | 212 | 0.34 | 0.16 | 0.18 |
| Fe_4_-N-C@TABOH | 502 | 248 | 0.39 | 0.13 | 0.26 |
| Fe_8_-N-C@TABOH | 365 | 163 | 0.26 | 0.10 | 0.16 |
| Fe_4_-N-C | 516 | 118 | 0.35 | 0.27 | 0.08 |

Table S2 Element contents of N, C, O and Fe in Fe*_x_*-N-C@TABOH and Fe_4_-N-C catalysts^a^

| Samples | Fe  (at.%) | N  (at.%) | C  (at.%) | O  (at.%) | Pyrrolic N (%) | Fe-N*_x_*  N (%) | Graphitic N (%) | Pyridinic N (%) |
| --- | --- | --- | --- | --- | --- | --- | --- | --- |
| Fe_1_-N-C@TABOH | 0.68 | 12.16 | 79.02 | 8.14 | 11.49 | 20.63 | 9.94 | 50.01 |
| Fe_2_-N-C@TABOH | 0.92 | 12.22 | 78.57 | 8.29 | 11.57 | 21.23 | 9.82 | 50.22 |
| Fe_4_-N-C@TABOH | 1.36 | 12.33 | 78.28 | 8.03 | 12.06 | 22.64 | 11.13 | 47.34 |
| Fe_8_-N-C@TABOH | 1.71 | 11.85 | 78.12 | 8.32 | 12.67 | 20.57 | 9.48 | 50.86 |
| Fe_4_-N-C | 1.67 | 11.36 | 78.52 | 8.45 | 12.84 | 19.45 | 9.82 | 49.09 |

^a^ Obtained from XPS analysis

Table S3 Electrochemical performance test of as prepared Fe*_x_*-N-C@TABOH catalysts

| Samples | Power density (mW·m^-2^) | Cell voltage output (V) | Onset potential (V vs RHE) | Half-wave potential  (V vs RHE ) | R_ct_ (Ω) |
| --- | --- | --- | --- | --- | --- |
| Fe_1_-N-C@TABOH | 582.7±6.1 | 0.62±0.02 | 0.81 | 0.66 | 15.2 |
| Fe_2_-N-C@TABOH | 758.3±3.6 | 0.64±0.01 | 0.83 | 0.68 | 13.7 |
| Fe_4_-N-C@TABOH | 830.1±5.2 | 0.67±0.01 | 0.86 | 0.71 | 9.7 |
| Fe_8_-N-C@TABOH | 465.0±3.9 | 0.60±0.02 | 0.82 | 0.67 | 16.8 |
| 20% Pt/C | 933.8±6.2 | 0.69±0.03 | 0.87 | 0.71 | 13.5 |
| Fe_4_-N-C | 626.5±4.0 | 0.61±0.02 | 0.82 | 0.67 | 13.3 |

Table S4 Electrochemical double-layer capacitance (C_dl_) and active surface area (ECSA)

of Fe*_x_*-N-C@TABOH and Fe_4_-N-C catalysts

| Samples | C_dl_ (mF·cm^-2^) | ECSA (cm^2^) |
| --- | --- | --- |
| Fe_1_-N-C@TABOH | 4.09 | 21.10 |
| Fe_2_-N-C@TABOH | 4.94 | 25.48 |
| Fe_4_-N-C@TABOH | 6.62 | 34.15 |
| Fe_8_-N-C@TABOH | 3.77 | 19.45 |
| Fe_4_-N-C | 4.69 | 24.19 |

Table S5 EXAFS fitting parameters at the Fe K-edge for various samples (*Ѕ*_0_^2^=0.77)

|  | Shell | N^a^ | R(Å)^b^ | σ^2^(Å^2^)^c^ | ΔE_0_(eV)^d^ | R factor(%)^e^ |
| --- | --- | --- | --- | --- | --- | --- |
| Fe foil | Fe-Fe | 8* | 2.47±0.01 | 0.0052 | 5.7±1.5 | 0.0068 |
|  | Fe-Fe | 6* | 2.85±0.01 | 0.0059 |  |  |
| FePc | Fe-N | 4.7±0.3 | 1.93±0.01 | 0.0017 | 8.7±1.4 | 0.0109 |
|  | Fe-C | 6.3±0.9 | 2.93±0.02 | 0.0003 |  |  |
| F_4_-N-C@TABOH | Fe-N | 4.8±0.7 | 1.95±0.01 | 0.0105 | 2.5±1.9 | 0.0083 |
|  | Fe-Fe | 4.4±0.2 | 2.50±0.01 | 0.0085 |  |  |
| F_4_-N-C | Fe-N | 4.0±0.7 | 1.89±0.01 | 0.0190 | -1.2±1.4 | 0.0130 |
|  | Fe-Fe | 5.8±0.3 | 2.49±0.01 | 0.0103 |  |  |

*^a^N*: coordination numbers; *^b^R*: bond distance; *^c^σ*^2^: Debye-Waller factors; *^d^* Δ*E*_0_: the inner potential correction. *R* factor: goodness of fit.

Table S6 Mössbauer spectrum results of Fe_4_-N-C@TABOH and Fe_4_-N-C

|  | | IS(mm/s) | QS(mm/s) | H(T) | Fe_4_-N-C@TABOH | | Fe_4_-N-C | |
| --- | --- | --- | --- | --- | --- | --- | --- | --- |
|  |  |  |  |  | Γ(mm/s) | Area(%) | Γ(mm/s) | Area(%) |
| Singlet | | -0.26 | — | — | 0.51 | 36.0 | 0.54 | 19.0 |
| Sextet | | 0.07 | -0.03 | 21.24 | 1.15 | 24.6 | 0.80 | 51.3 |
| Doublet | D1 | 0.32 | 0.91 | — | 0.76 | 25.2 | 0.88 | 21.5 |
|  | D2 | 0.20 | 2.54 | — | 0.88 | 4.6 | 0.88 | 1.4 |
|  | D3 | 0.83 | 1.93 | — | 0.19 | 0.6 | 0.19 | 0.3 |
|  | D4 | 0.24 | 1.45 | — | 0.88 | 5.8 | 0.88 | 3.7 |
|  | D5 | 0.61 | 3.36 | — | 0.88 | 3.0 | 0.88 | 2.0 |
|  | D6 | 0.37 | 1.39 | — | 0.19 | 0.2 | 0.45 | 0.8 |

Table S7 Comparison of ORR activity between Fe_4_-N-C@TABOH with

other catalysts reported in the literatures

| Catalyst | *E_onset_*  [V vs RHE] | *E_1/2_*  [V vs RHE] | Reference |
| --- | --- | --- | --- |
| **Fe_4_-N-C@TABOH** | **0.86** | **0.71** | **This work** |
| Fe(Zn)-N-C | 0.83 | 0.67 | [3] |
| Fe_0.025_-N@MOF | 0.75 | 0.57 | [4] |
| Fe-SAs/S,N-C/rGO | 0.84 | 0.60 | [5] |
| FeSNC-3 | 0.89 | 0.69 | [6] |
| 5%Fe-N/C | 0.82 | 0.64 | [7] |
| Fc@Fe-NHCS | 0.87 | 0.76 | [8] |
| Fe_3_Co_1_-700 | 0.91 | 0.66 | [9] |
| FeP-900 | 0.85 | 0.70 | [10] |
| Fe-NpC | 0.87 | 0.71 | [11] |
| Co_3_Fe_7_-Fe_3_C/HNC | 0.78 | 0.64 | [12] |
| Fe-N-C-NH_3_ | 0.86 | 0.65 | [13] |

Table S8 Comparison of Fe_4_-N-C@TABOH with other reported ORR catalysts towards power density in MFC

| Catalysts | Preparation conditions | Power density (mW/m^2^) | Reference |
| --- | --- | --- | --- |
| **Fe_4_-N-C@TABOH** | **900 °C, Ar** | **830.1** | **This work** |
| Cu/Fe/N-C#3 | 900 °C /N_2_ | 423.0 | [14] |
| Fe(0.3)@N–C | 700 °C, N_2_ | 604.6 | [15] |
| Fe-N-S/C | 600 °C/N_2_ | 923.0 | [16] |
| Mn-Fe@g-C_3_N_4_ | 700 °C/N_2_ | 413.0 | [17] |
| Fe/Fe_3_C/NPGC–650 | 650 °C /N_2_ | 1323 | [18] |
| N_3_/Fe/C-Pt | 900 °C /N_2_ | 504.0 | [19] |
| GCN-Co@CoO | 800 °C /N_2_ | 611.0 | [20] |
| NiFe-LDH@Co_3_O_4_ | 130°C, hydrothermal approach | 467.4 | [21] |

**References**

[1] C.C.L. McCrory, S.H. Jung, J. C. Peters, T. F. Jaramillo, *J. Am. Chem. Soc.*, **2013**, *135*, 16977-16987.

[2] J.K. Nùrskov, F. Abild-Pedersen, F. Studt, B. Thomas. *Proc. Natl. Acad. Sci. USA*, **2011**, *108*, 937-943.

[3] Y. Ma, Y. Xiao, Y. Ge, D. Gao, Y. Zhang, Z. Li, Y. Han, *J. Mater. Chem. A*, **2024**, *12*, 2004.

[4] H. Zhang, Y. Wang, T. Wu, J. Yu, S. R. B. Arulmani, W. Chen, L. Huang, M. Su, J. Yan, X. Liu, *J. Alloys Compd.*, **2023**, *944*, 169039.

[5] L. Li, N. Li, J.-W. Xia, S.-L. Zhou, X.-Y. Qian, F.-X. Yin, G.-H. Dai, G.-Y. He, H.Q. Chen, *Nano Research*, **2023**, *16*, 9416.

[6] B. Li, Q. Li, X. Wang, Environ. Res. **2023**, 228, 115808.

[7] L.Q. Yu, H. Wang, S.-L. Chen, T.-E. Wen, B.-C. Huang, R.C. Jin, *Chin. Chem. Lett.*, **2023**, *34*, 107236.

[8] K. Sheng, J. Li, G. Li, J. Hao, Y. Wang, Y. Liu, Y. Liu, Q. Yi, W. Li, *Appl. Surf. Sci.*, **2022**, *601*, 154221.

[9] S. Zhuang, B. Li, X. Wang, *Environ. Res.*, **2023**, *216*, 114542.

[10] X. Luan, W. Bian, D. Guo, B. Wang, W. Tan, F. Peng, B. Zhou, *New J. Chem.*, **2023**, *47*, 1625.

[11] X. Wang, H. Zhang, J. Ye, B. Li, *J. Power Sources*, **2023**, *556*, 232434.

[12] M. Jiang, C. Fu, R. Cheng, T. Liu, M. Guo, P. Meng, J. Zhang, B. Sun, *Chem. Eng. J.*, **2021**, *404*, 127124.

[13] Z. Chen, D. Zhao, C. Chen, Y. Xu, C. Sun, K. Zhao, M. Arif Khan, D. Ye, H. Zhao, J. Fang, X. Andy Sun, J. Zhang, *J. Colloid Interface Sci.*, **2021**, *582*, 1033.

[14] H.Q. Wang, L.L. Wei, B.R. Duan, J.T. Liu, J.Q. Shen, *J. Electroanal. Chem.*, **2020**, *877*, 114556.

[15] Y.Y. Cui, N. Jiang, S.J. Shi, C. Jia, B. Jiang, *Int. J. Energ. Res.*, **2022**, *46*(15), 23234-23243.

[16] P.Y. Jiang, Z.H. Xiao, S.H. Li, Z.N. Luo, R. Qiu, H.X. Wu, N. Li, Z.Q. Liu, *J. Power Sources*, **2021**, *512*, 230491.

[17] C. Zhang, Y.C. Wang, B. An, R.Y. Huang, C. Wang, Z.Y. Zhou, W.B. Lin, *Adv. Mater.*, **2017**, *29*, 1-7.

[18] V. M. Ortiz-Martínez, M.J. Salar-García, K.Touati, F.J. Hernández-Fernández, A.P. Ríos, F. Belhoucine, A.A. Berrabbah, *Energy*, **2016**, *113*, 1241-1249.

[19] Q.X. Lai, L.R. Zheng, Y.Y. Liang, J.P. He, J.X. Zhao, J.H. Chen, *ACS Catal.*, **2017**, *7*, 1655-1663.

[20] L. Tan, Q. Pan, X. Wu, N. Li, J. Song, Z. Liu, *ACS Sustainable Chem. Eng.*, **2019**, *7*, 6335-6344.

[21] H. Wang, L.Wei, J. Liu, J. Shen, *Int. J. Hydrogen Energ.*, **2020**, *45*, 4481-4489.
